# Identifying diagnostic indicators for type 2 diabetes mellitus from physical examination using interpretable machine learning approach

**DOI:** 10.3389/fendo.2024.1376220

**Published:** 2024-03-18

**Authors:** Xiang Lv, Jiesi Luo, Wei Huang, Hui Guo, Xue Bai, Pijun Yan, Zongzhe Jiang, Yonglin Zhang, Runyu Jing, Qi Chen, Menglong Li

**Affiliations:** ^1^ College of Chemistry, Sichuan University, Chengdu, China; ^2^ Basic Medical College, Southwest Medical University, Luzhou, China; ^3^ Department of Endocrinology and Metabolism, The Affiliated Hospital of Southwest Medical University, Luzhou, China; ^4^ Metabolic Vascular Disease Key Laboratoryof Sichuan Province, The Affiliated Hospital of Southwest Medical University, Luzhou, China; ^5^ Department of Pharmacy, The Affiliated Hospital of North Sichuan Medical College, Nanchong, China; ^6^ School of Cyber Science and Engineering, Sichuan University, Chengdu, China; ^7^ Department of Nursing, The Affiliated Hospital of Southwest Medical University, Luzhou, China; ^8^ School of Nursing, Southwest Medical University, Luzhou, China

**Keywords:** diabetes, diabetes diagnosis, diabetic prediction, diagnostic indicator, health informatics, interpretable machine learning

## Abstract

**Background:**

Identification of patients at risk for type 2 diabetes mellitus (T2DM) can not only prevent complications and reduce suffering but also ease the health care burden. While routine physical examination can provide useful information for diagnosis, manual exploration of routine physical examination records is not feasible due to the high prevalence of T2DM.

**Objectives:**

We aim to build interpretable machine learning models for T2DM diagnosis and uncover important diagnostic indicators from physical examination, including age- and sex-related indicators.

**Methods:**

In this study, we present three weighted diversity density (WDD)-based algorithms for T2DM screening that use physical examination indicators, the algorithms are highly transparent and interpretable, two of which are missing value tolerant algorithms.

**Patients:**

Regarding the dataset, we collected 43 physical examination indicator data from 11,071 cases of T2DM patients and 126,622 healthy controls at the Affiliated Hospital of Southwest Medical University. After data processing, we used a data matrix containing 16004 EHRs and 43 clinical indicators for modelling.

**Results:**

The indicators were ranked according to their model weights, and the top 25% of indicators were found to be directly or indirectly related to T2DM. We further investigated the clinical characteristics of different age and sex groups, and found that the algorithms can detect relevant indicators specific to these groups. The algorithms performed well in T2DM screening, with the highest area under the receiver operating characteristic curve (AUC) reaching 0.9185.

**Conclusion:**

This work utilized the interpretable WDD-based algorithms to construct T2DM diagnostic models based on physical examination indicators. By modeling data grouped by age and sex, we identified several predictive markers related to age and sex, uncovering characteristic differences among various groups of T2DM patients.

## 1 Introduction

Type 2 diabetes mellitus (T2DM) is the most common type of diabetes mellitus (DM), whose pathogenesis is that the cells in the body are not sensitive to insulin, meaning they do not respond to insulin (1). A longer disease duration of diabetes often leads to a variety of complications, such as retinopathy (2), cardiovascular disease, stroke (3, 4), and diabetic foot (5). It is estimated that about half of T2DM patients do not know they have diabetes (44.7%) (6). Therefore, screening for T2DM is essential to prevent or delay complications, avoid premature death, and improve quality of life.

Manual review of a large amount of clinical data is time-consuming and laborious, and missed diagnosis will be inevitable (6, 7). Thus, leveraging machine learning for T2DM screening has emerged as a notable approach in auxiliary diagnostics, enhancing both the accuracy and efficiency of diagnoses. Currently, machine learning models such as the random forest (RF) (8–10), support vector machine (SVM) (8, 11), logistic regression (LR) (11–13), and eXtreme gradient boosting (XGBoost) (9, 14) have been developed for constructing accurate system of T2DM prediction. Some studies have also employed machine learning techniques to identify indicators associated with T2DM, such as the white blood cell (WBC) (15), urinary and dietary metal exposure (16) and serum calcium (17). These works demonstrate the effectiveness of machine learning in predicting T2DM and identifying relevant indicator information.

For the construction of T2DM diagnostic models, the existing problems are as follows: (I) The effective extraction of T2DM diagnostic indicators through machine learning often relies on their interpretability (10, 12, 18–23). However, some of the current work lacks evaluation of important indicators, and some rely on third-party tools such as Shapley Additive exPlanations (SHAP) and Local Interpretable Model-agnostic Explanations (LIME) (8, 9, 11), which may bring potential deviation in clinical understanding (24). (II) The clinical indicators and datasets are critical to whether a model can be used in practice. At present, the frequently used diabetes dataset public like PIMA Indian dataset contains only 8 clinical indicators (25), and unconventional indicators using in some works are often difficult to obtain in community hospitals. Therefore, it is valuable to use physical examination indicators for T2DM prediction. (III) The problem of missing values in EHRs is unavoidable during data analysis. Currently, methods based on data imputation often require exhaustive searching (26). How to handle these missing values reasonably and efficiently is a matter that needs consideration.

To this end, we introduced three weighted diversity density (WDD)-based algorithms with a focus on intrinsic interpretability, which two of the algorithms could ‘tolerate’ missing value by adding penalty terms. By applying these algorithms to physical examination data for T2DM, we identified several clinical indicators related to T2DM diagnosis, including age-related markers like glomerular filtration rate (GFR) and triglycerides (TG). Additionally, by analyzing the model’s internal parameters, we can gain a better understanding of the clinical indicators the model relies on for predictions, without the need for third-party interpretability tools.

## 2 Materials and methods

### 2.1 Dataset summary

**Figure 1:**
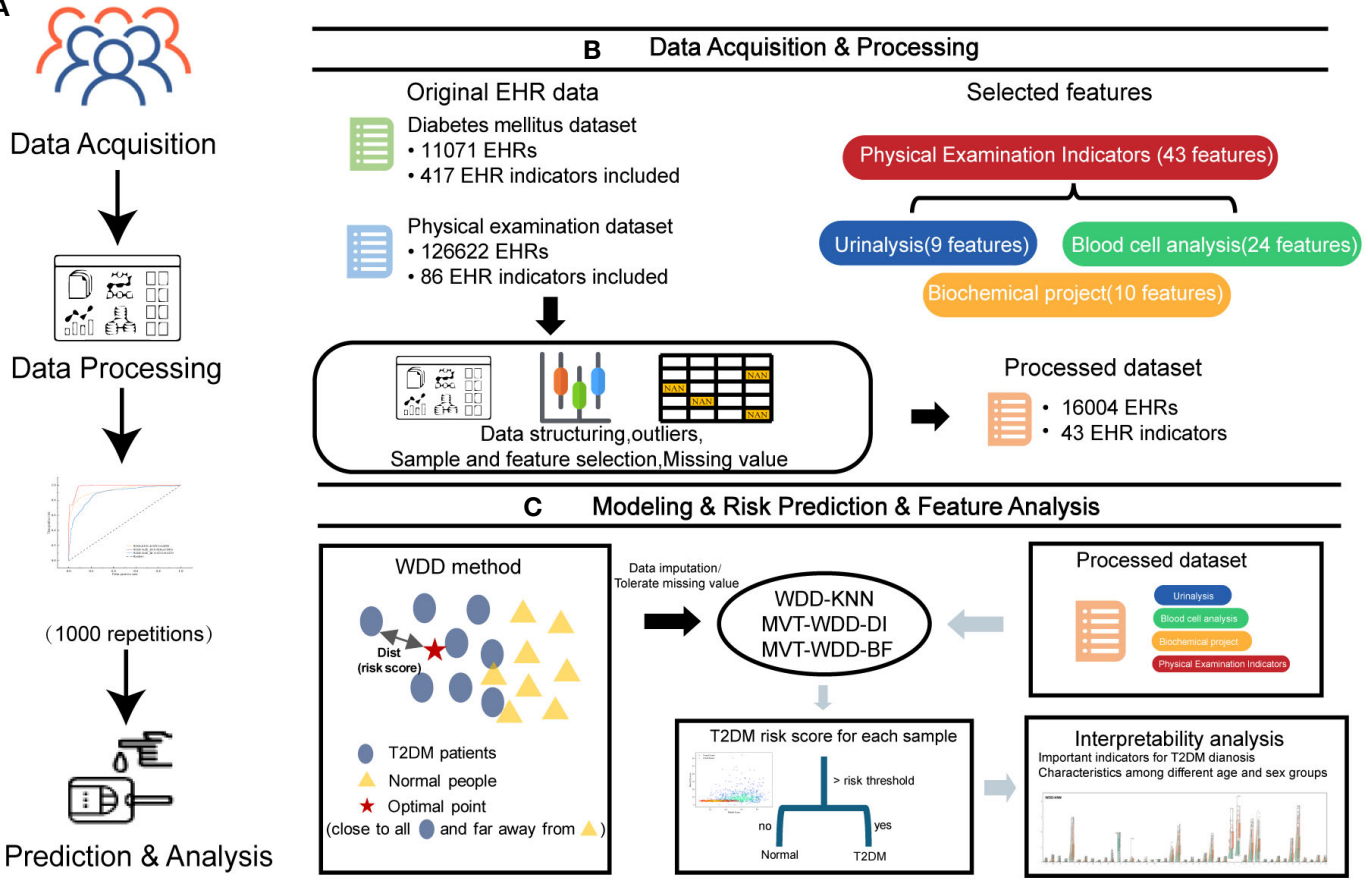
Overview of the study design. **(A)** The workflow of this work. **(B)** The first two steps in A, 11071 T2DM electronic health records (EHRs) and 126622 physical examination EHRs were collected. After preprocessing, 16004 EHRs were selected to build models for T2DM prediction. **(C)** The last two steps in A and the basic principle of the weighted diversity density method.

**Figure 2:**
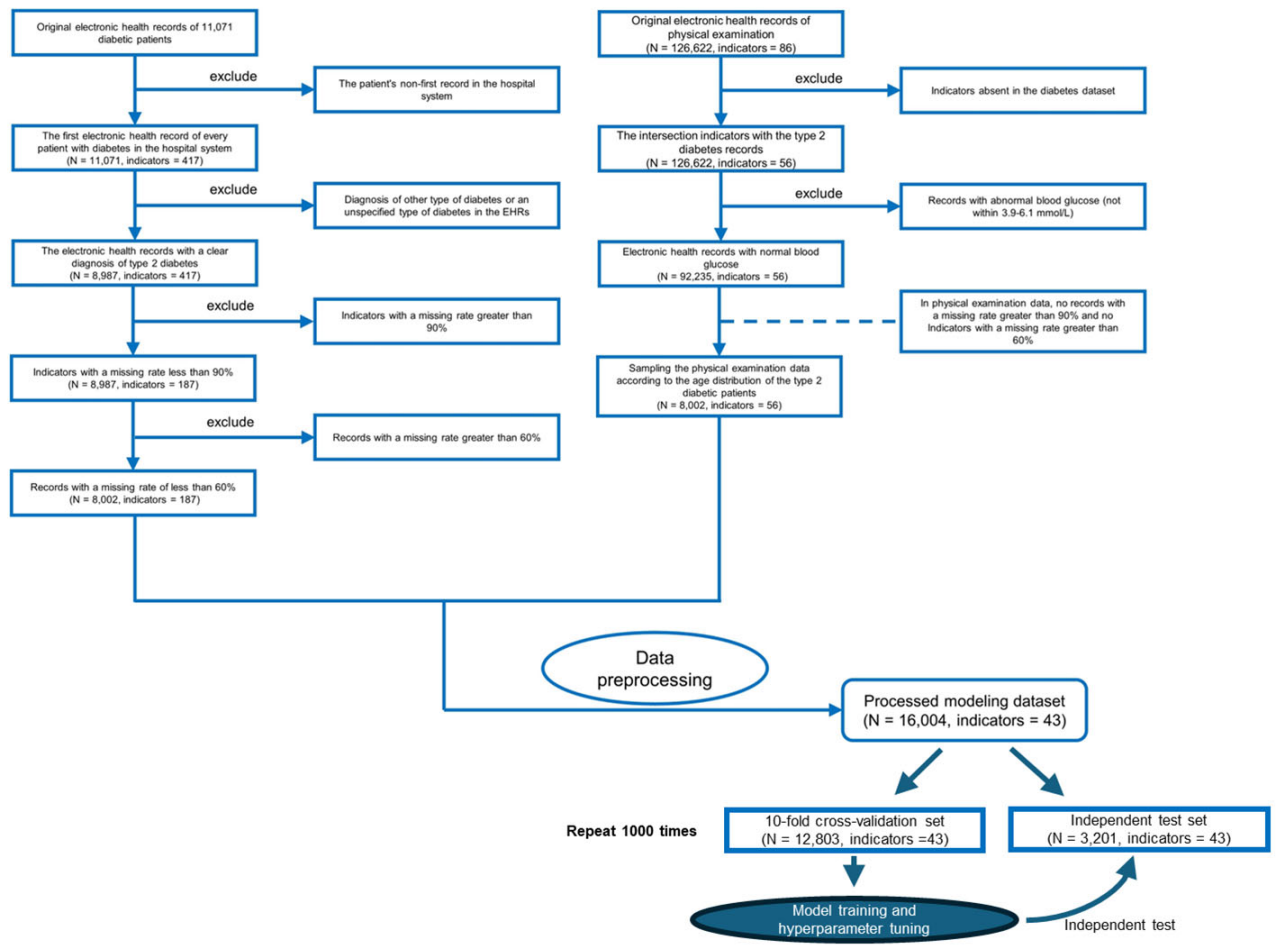
Flowchart of inclusion and exclusion criteria for the study populations of patients with type 2 diabetes mellitus (T2DM) and the physical examination population. We only use the first electronic health records for each patient in the hospital system.

All electronic health record (EHR) data came from the Affiliated Hospital of Southwest Medical University. A total of 16,004 EHRs and 43 usable physical examination indicators were screened out, of which half explicitly contained information about a confirmed T2DM diagnosis (**Figures 1**, **2**; **Table 1**). In order to capture characteristics of early-stage T2DM, the EHRs of T2DM patients were limited to their first record in the hospital system. The physical examination indicators could be divided into three categories: routine urine indicators (9 indicators), blood cell analysis indicators (24 indicators), and biochemical indicators (10 indicators) (**Figures 1**, **2**), the name and the abbreviation of the indicators were shown in **Supplementary Table S1**.

**Table 1:** Characteristics of the study population.

	Normal people	T2DM patients
TC, mmol/L	4.96 (0.932)	4.63 (1.393)
AST, U/L	24.58 (12.877)	25.20 (37.516)
GFR, mL/min	94.01 (14.660)	87.98 (29.298)
Crea, umol/L	68.48 (20.157)	86.93 (80.094)
HDL-C, mmol/L	1.40 (0.368)	1.14 (0.369)
TG, mmol/L	1.61 (1.313)	2.28 (2.377)
LDL-C, mmol/L	3.09 (0.900)	2.71 (1.014)
ALT, U/L	24.78 (19.111)	26.82 (32.369)
GGT, U/L	30.62 (40.086)	49.49 (110.088)
AST/ALT	1.19 (0.547)	1.18 (0.969)
MUCUS,/uL	12.88 (18.193)	2.75 (12.213)
BACT,/uL	21.38 (366.085)	1998.54 (7639.814)
EC,/uL	6.81 (25.506)	5.28 (14.011)
BLD	0.31 (0.628)	0.27 (0.603)
U-SG	1.02 (0.006)	1.02 (0.008)
U-pH	5.88 (0.729)	5.73 (0.719)
Crystal,/uL	6.09 (37.158)	23.45 (178.737)
RBC-Urine,/uL	7.29 (165.853)	20.96 (267.325)
WBC-Urine,/uL	14.43 (140.372)	61.44 (640.552)
NEU, 10E9/L	3.64 (1.290)	5.50 (3.591)
NEU-R, %	59.31 (8.511)	68.59 (11.639)
PCT, %	0.22 (0.051)	0.24 (0.086)
PDW, %	16.15 (1.010)	15.48 (2.733)
PLT, 10E9/L	210.83 (56.276)	206.96 (80.247)
MPV, fL	10.74 (1.412)	11.44 (1.400)
HGB, g/L	142.27 (15.014)	126.97 (21.740)
EOS, 10E9/L	0.16 (0.161)	0.13 (0.172)
EOS-R, %	2.62 (2.317)	1.99 (2.336)
BASO, 10E9/L	0.03 (0.018)	0.02 (0.020)
BASO-R, %	0.53 (0.283)	0.24 (0.258)
LYM, 10E9/L	1.88 (0.617)	1.57 (0.665)
LYM-R, %	31.65 (7.991)	23.10 (10.179)
HCT	0.44 (0.041)	0.39 (0.063)
RDW-SD, fL	43.18 (2.994)	42.91 (4.282)
RDW-CV, %	13.15 (0.975)	13.34 (1.305)
RBC, 10E12/L	4.66 (0.489)	4.33 (0.732)
MCHC, g/L	325.53 (8.441)	328.30 (14.113)
MCH, pg	30.62 (2.417)	29.40 (2.533)
MCV, fL	94.00 (6.445)	89.55 (6.784)
MONO, 10E9/L	0.35 (0.122)	0.45 (0.257)
MONO-R, %	5.88 (1.491)	6.12 (2.300)
P-LCR	31.37 (9.845)	36.20 (10.018)
WBC, 10E9/L	6.08 (2.075)	7.68 (3.860)

Data are mean (standard deviation).

For these datasets divided according to the physical examination items, to facilitate their description here, we chose some standardized abbreviations: Whole physical examination indicators dataset (PEI dataset), Blood cell analysis dataset (BCA dataset), Urinalysis dataset (Uri dataset), Biochemical dataset (BioChem dataset).

### 2.2 Data preprocessing

On the collected data, three steps were performed before modelling:

(1) First, we retained only the physical examination indicators for exploring the association of these indicators with T2DM diagnosis. In total, 181 features, of which 52 are physical examination features, remained afterwards.
(2) The features were normalized by $X_{ij}^{\prime }=\frac{\left(X_{ij}-\mu_{j}\right)}{\sigma_{j}}$ , where $\mu_{j}$ and $\sigma_{j}$ are the average and standard deviation of the i th record, respectively. To avoid the influence from outliers, a featurewise box-plot analysis was performed to remove the features located outside of [Q1−1.5IQR,Q3+1.5IQR] when calculating $\mu_{j}$ and $\sigma_{j}$ . These outliers were replaced by null values. Any feature with a standard deviation of 0 (that is, the feature value is the same in all samples) was deleted, leaving 43 dimensions of physical examination features.
(3) When using the WDD-KNN algorithm for modelling, we used the K-nearest neighbour (KNN) imputer to impute the missing values of the data (n_neighbours = 15) and then normalized the processed data again in step (2).

The missing rate of each feature is shown in **Supplementary Table S3**. When using the MVT-WDD-DI and MVT-WDD-BF algorithms for modelling, we kept the missing value of each dimension feature of positive and negative samples consistent to eliminate bias. (Details in the **Supplementary Note 4**: Biased distribution misleading model using features).

### 2.3 Preliminary of weighted diversity density algorithm

The WDD algorithm, initially proposed by Maron for multi-instance learning problems (27), utilizes radial basis distance metrics to measure classification probabilities. We developed three new algorithms based on WDD in this work. Its non-linear nature and transparent framework make it particularly suitable for the medical field, where high interpretability in models is essential. Building upon the WDD framework, we have made enhancements to adapt it for medical classification problems, ensuring it can effectively handle missing values. Additionally, another reason for selecting the WDD method is its capability to provide a risk score for each sample, similar to the diagnostic approach of a clinical physician. This is achieved through its internal distance function, rather than merely outputting a categorical label. By decomposing the distance function at the feature level, we are further able to extract the feature-based criteria on which the model relies. This significantly enhances the transparency of the model. Unlike traditional models that offer limited insight into their decision-making process, WDD allows for a deeper understanding of how and why certain diagnostic conclusions are reached. This alignment with clinical practices not only aids in the interpretability of the model but also fosters greater trust and reliability in its application in medical settings. The ability to dissect the model’s reasoning at a feature level offers invaluable insights into the diagnostic criteria, bridging the gap between machine learning outputs and clinical decision-making.

The major principle of the WDD algorithm (**Figure 1C**) is to find an optimal point $x=\left\{x_{1},x_{2},\ldots ,x_{f}\right\}$ in the data space that maximizes the probability density that positive samples ( $b_{i}^{+}$ = $\left\{b_{i1}^{+},b_{i2}^{+},\ldots ,b_{if}^{+}\right\}$ ) are near this point and minimizes the probability density that negative samples ( $b_{i}^{-}$ = $\left\{b_{i1}^{-},b_{i2}^{-},\ldots ,b_{if}^{-}\right\}$ ) are near it, where i is the index of the sample, f is the number of features. The modified WDD algorithm can be represented as:


(1) $$\arg\underset{x}{\max}\left(\prod_{i}\mathrm{Pr}(x=t\;|\;b_{i}^{+})\prod_{i}\mathrm{Pr}(x=t\;|\;b_{i}^{-})\right)\;$$

t is the target point, $\mathrm{Pr}(x=t\;|\;b_{i}^{+})$ is the probability density that positive samples are near this point, and $\mathrm{Pr}(x=t\;|\;b_{i}^{-})$ is the probability density of negative samples. The implicit functions could be given as:


(2) $$\mathrm{Pr}(x=t\;|\;b_{i}^{+})=\exp\left(-Dist\left(b_{i}^{+},x\right)\right)$$

(3) $$\mathrm{Pr}(x=t\;|\;b_{i}^{-})=1-\exp\left(-Dist\left(b_{i}^{-},x\right)\right)$$

where distance function (Dist) for both positive and negative samples is defined as the sum of weighted squares:


(4) $$Dist\left(b_{i},x\right)=\sum_{k}Dist_{k}$$

(5) $$Dist_{k}=s_{k}{\left(b_{ik}-x_{k}\right)}^{2}$$

where k is the index of the feature.

The formulas yield a function to measure an optimal point based on both positive and negative samples, and the position of the point can be optimized during a deep learning process.

In this work, based on the idea of Maron’s work, we modified the distance function to achieve our predictive goals. One of the algorithms first imputes the data with missing values by k-nearest neighbour (KNN) and then uses WDD for prediction. The other two algorithms, named MVT-WDD-DI and MVT-WDD-BF, do not need to impute data but use penalty mechanisms for missing value tolerance.

### 2.4 Our proposed modified WDD algorithm

As mentioned above, we modified the WDD algorithm proposed in Maron’s work to improve the algorithms’ ability to handle data containing missing values. The modifications are introduced in the following subsections:

#### 2.4.1 WDD-KNN

The WDD-KNN algorithm uses data imputation by k-nearest neighbor (KNN) as input and then uses the modified WDD algorithm for modelling. Since all the missing values are imputed by KNN, we only modified Equations (2) and (3) by adding a hyperparameter γ to make the calculation more flexible:


(6) $$\mathrm{Pr}(x=t\;|\;b_{i}^{+})=\exp\left(-\gamma Dist\left(b_{i}^{+},x\right)\right)$$

(7) $$\mathrm{Pr}(x=t\;|\;b_{i}^{-})=1-\exp\left(-\gamma Dist\left(b_{i}^{-},x\right)\right)$$

Using WDD-KNN, we can classify a dataset containing missing values, but it still needs a step for missing value filling. This step limits the process of prediction. When given a new sample that contains a missing value, we need a dataset for imputing the missing value more than the trained model parameters. Therefore, we developed two missing value tolerant (MVT) algorithms for our predictive goals.

#### 2.4.2 MVT-WDD-DI

We developed MVT-WDD-DI by adding penalty term for Equations (6) and (7) using division (DI) to handle missing values and finally obtain Equations (8) and (9):


(8) $$\mathrm{Pr}(x=t\;|\;b_{i}^{+})=\exp\left(-\gamma \left(\frac{Dist\left(b_{i}^{+},x\right)}{{\left(f-f_{miss}\right)}^{\delta }}\right)\right)$$

(9) $$\mathrm{Pr}(x=t\;|\;b_{i}^{-})=1-\exp\left(-\gamma \left(\frac{Dist\left(b_{i}^{-},x\right)}{{\left(f-f_{miss}\right)}^{\delta }}\right)\right)$$

where f is the number of features and $f_{miss}$ is the number of missing values. Since the modification will influence the calculation of Equations (6) and (7), we added a rule to Equation (5): $if\;b_{ik}\;is\;missing,\;b_{ik}-x_{k}=0$ . We also added a hyperparameter δ for adjusting the value of the penalty term. The concept behind this modification is to diminish the influence of samples containing missing values. Specifically, when a sample has numerous missing values, indicating lower data quality, we increase the penalty term to reduce its distance metric value. This approach, during the optimization process, results in the target point relying less on these data segments. The penalty term can reduce the diversity density according to the number of missing features and will not influence a sample containing all the features.

#### 2.4.3 MVT-WDD-BF

In addition to using division to penalize missing values, we also tried to design another method by ignoring the missing feature (BF) of a sample. To make the idea work, we modified Equations (2) through (5) of the original WDD algorithm. First, Equations (4) and (5) were modified to


(10) $$Dist^{\prime }\left(b_{i},x,\gamma ,\lambda \right)=\sum_{k}Dist_{k}^{\prime^{\lambda }}\;$$

(11) $$Dist_{k}^{\prime }=\exp\left(-\gamma s_{k}\left|b_{ik}-x_{k}\right|\right)$$

In Equation (11), $if\;b_{ik}\;is\;missing,\;\exp\left(-\gamma s_{k}\left|b_{ik}-x_{k}\right|\right)=0$ . This modification allows the algorithm to ‘ignore’ a missing feature when calculating. However, a reduced number of features will increase the diversity density based on the analysis of monotonicity. We modified Equations (2) and (3) to solve this problem:


(12) $$\mathrm{Pr}(x=t\;|\;b_{i}^{+})=\;1-\exp(-\mathrm{Pr}(x=t\;|\;b_{i}^{+}))$$

(13) $$\mathrm{Pr}(x=t\;|\;b_{i}^{-})=\exp\left(-\mathrm{Pr}(x=t\;|\;b_{i}^{-}\right))$$

In Equations (12) and (13), we changed the monotonicity by removing the minuend Equations ‘1’ in the production term and added Euler’s number as the base for scaling an excessively large negative number to the range (0,1) after a practical test.

### 2.5 Model setup

Since the goal of WDD is to find a point to maximize the diversity density, we used a deep learning framework for implementation. We used the Adam optimizer to solve the optimal problem, and the loss function of the three algorithms is shown in Equation (14):


(14) $$loss=\;-log\left(\prod_{i}\mathrm{Pr}(x=t\;|\;b_{i}^{+})\prod_{i}\mathrm{Pr}(x=t\;|\;b_{i}^{-})\right)$$

### 2.6 Model inference

After training, we obtained the optimal position $x_{target}$ from the dataset, which optimized Equation (1). Thus, all the samples could be classified by calculating the maximum diversity density from its instances:


(15) $$DD_{i}=\exp\left(-\text{Dist}\left(b_{i},x\right)\right)$$

Using Equation (15) calculated diversity density, a sample could be assigned a label by setting a threshold. In this work, we used the method of analysing the ROC curve from the training set. First, we calculated all the diversity density values of the samples in the training dataset. Then, we calculated the false positive rate (FPR) and true positive rate (TPR), also called recall, under several different cut-off points and selected the best cut-off as the threshold when TPR-FPR reached its maximum The details are given in Equations (16) - (18):


(16) $$\text{threshold }=\arg\underset{cutoff}{\max}(TPR-FPR)\;\;\;$$

(17) $$TPR=\;\frac{TP\;}{\left(TP\;+\;FN\right)}\;$$

(18) $$FPR=\;\frac{FP\;}{\left(TN\;+\;FP\right)}$$

Similar to many other works, we employed the area under the receiver operating characteristic curve (AUC), accuracy (ACC), precision, recall, and F1 score as the metrics, calculated as Equations (19) - (22):


(19) $$ACC=\;\frac{TP+TN\;}{\left(TP+TN\;+\;FP+FN\right)}$$

(20) $$Precision=\;\frac{TP\;}{\left(TP\;+\;FP\right)}$$

(21) $$Recall=\;\frac{TP\;}{\left(TP\;+\;FN\right)}$$

(22) $$F1\;score=\;2*\frac{Precision*Recall}{Precision+Recall}$$

where TP, TN, FP and FN represent the number of true positives, true negatives, false positives and false negatives, respectively.

Additional details about the method, such as parameter tuning and training process, are provided in Supplemental information.

### 2.7 Basic workflow

In this study, we optimized the WDD model using gradient descent to identify an optimal point that is close to the distribution center of data from T2DM patients and far from the distribution center of data from normal individuals (**Figure 1C**). The Dist function is engineered to directly reflect the T2DM risk score, enabling the model to predict an input sample as T2DM if its risk score exceeds the risk threshold which is learned by the model. Through the analysis of learnable parameters within the Dist function, we have identified key features. Utilizing this characteristic, we have been able to uncover significant features across different age and gender groups, enhancing our understanding of T2DM risk factors.

## 3 Result

In the results section, we first introduce the performance scores of the model, confirming the consistency of its performance by repeating the modeling process 1000 times. More importantly, our discussion centers on the model’s transparency and interpretability, aimed at extracting effective clinical information internally. This includes the identification of key diagnostic indicators and the interpretation of the associations between model parameters and prediction result. These sections together demonstrate the model’s transparency and potential clinical utility, contributing useful perspectives for T2DM prediction and diagnosis.

### 3.1 Performance of the prediction model

To the above four datasets, we applied three weighted diversity density (WDD)-based algorithms to construct diagnostic prediction models. WDD-KNN refers to an algorithm using k-nearest neighbor (KNN) for imputing missing values. The two MVT-WDD algorithms denote missing value tolerant (MVT) algorithms with penalty terms, where ‘DI’ and ‘BF’ represent two different methods of penalization. A total of 12 models were obtained. The models were evaluated by 10-fold cross-validation and independent test with 1000 repetitions (**Figure 2**, cross-validation set: independent test set = 8:2, the independent test dataset was consistent for each repetition, details in **Supplementary Note 1**). The results are shown in **Table 2**, the best AUC achieved 0.9185 ( ± 0.0035) on the whole PEI dataset, which proves the accuracy of the model.

**Table 2:** Performance of algorithms on each dataset: Mean (Standard) of 1000 repetitions.

10-fold cross-validation					
PEI dataset					
	AUC	ACC	Precision	Recall	F1 score
WDD-KNN	0.9185 (0.0034)	0.8439 (0.0042)	0.8761 (0.0049)	0.8014 (0.0073)	0.8368 (0.0047)
MVT-WDD-DI	0.9130 (0.0088)	0.8404 (0.0103)	0.8726 (0.0106)	0.7973 (0.0130)	0.8329 (0.0111)
MVT-WDD-BF	0.8882 (0.0096)	0.8138 (0.0093)	0.8291 (0.0096)	0.7910 (0.0135)	0.8091 (0.0103)
BCA dataset					
	AUC	ACC	Precision	Recall	F1 score
WDD-KNN	0.8770 (0.0018)	0.7992 (0.0021)	0.8386 (0.0039)	0.7418 (0.0058)	0.7868 (0.0028)
MVT-WDD-DI	0.8530 (0.0045)	0.7763 (0.0044)	0.8097 (0.0062)	0.7233 (0.0093)	0.7635 (0.0054)
MVT-WDD-BF	0.8910 (0.0094)	0.8156 (0.0089)	0.8379 (0.0081)	0.7829 (0.0152)	0.8087 (0.0109)
Uri dataset					
	AUC	ACC	Precision	Recall	F1 score
WDD-KNN	0.8442 (0.0069)	0.7768 (0.0096)	0.7621 (0.0133)	0.8133 (0.0240)	0.7837 (0.0109)
MVT-WDD-DI	0.8985 (0.0074)	0.8580 (0.0096)	0.8463 (0.0109)	0.8763 (0.0126)	0.8604 (0.0101)
MVT-WDD-BF	0.6414 (0.0175)	0.6321 (0.0129)	0.6993 (0.0203)	0.4795 (0.0355)	0.5580 (0.0265)
BioChem dataset					
	AUC	ACC	Precision	Recall	F1 score
WDD-KNN	0.7360 (0.0016)	0.6766 (0.0021)	0.6900 (0.0040)	0.6432 (0.0092)	0.6651 (0.0039)
MVT-WDD-DI	0.7395 (0.0128)	0.6801 (0.0089)	0.7075 (0.0084)	0.6137 (0.0237)	0.6540 (0.0185)
MVT-WDD-BF	0.6127 (0.0212)	0.5987 (0.0136)	0.6172 (0.0258)	0.4924 (0.0430)	0.5389 (0.0376)
Independent test					
PEI dataset					
	AUC	ACC	Precision	Recall	F1 score
WDD-KNN	0.9276 (0.0089)	0.8554 (0.0112)	0.8888 (0.0142)	0.8128 (0.0198)	0.8489 (0.0125)
MVT-WDD-DI	0.9194 (0.0254)	0.8475 (0.0310)	0.8826 (0.0299)	0.8014 (0.0411)	0.8398 (0.0340)
MVT-WDD-BF	0.9071 (0.0214)	0.8296 (0.0235)	0.8429 (0.0280)	0.8110 (0.0298)	0.8263 (0.0243)
BCA dataset					
	AUC	ACC	Precision	Recall	F1 score
WDD-KNN	0.8870 (0.0057)	0.8106 (0.0072)	0.8469 (0.0098)	0.7551 (0.0200)	0.7982 (0.0099)
MVT-WDD-DI	0.8625 (0.0151)	0.7851 (0.0152)	0.8198 (0.0192)	0.7273 (0.0294)	0.7704 (0.0184)
MVT-WDD-BF	0.8988 (0.0326)	0.8226 (0.0301)	0.8467 (0.0309)	0.7850 (0.0482)	0.8140 (0.0362)
Uri dataset					
	AUC	ACC	Precision	Recall	F1 score
WDD-KNN	0.8224 (0.0207)	0.7569 (0.0252)	0.7621 (0.0426)	0.7406 (0.0893)	0.7463 (0.0359)
MVT-WDD-DI	0.8893 (0.0230)	0.8499 (0.0291)	0.8355 (0.0348)	0.8635 (0.0356)	0.8488 (0.0303)
MVT-WDD-BF	0.6304 (0.0581)	0.6306 (0.0396)	0.6863 (0.0649)	0.4670 (0.1243)	0.5433 (0.0891)
BioChem dataset					
	AUC	ACC	Precision	Recall	F1 score
WDD-KNN	0.7482 (0.0045)	0.6885 (0.0065)	0.6975 (0.0141)	0.6588 (0.0256)	0.6771 (0.0098)
MVT-WDD-DI	0.7464 (0.0456)	0.6862 (0.0300)	0.7098 (0.0255)	0.6186 (0.0841)	0.6573 (0.0684)
MVT-WDD-BF	0.6290 (0.0653)	0.6104 (0.0442)	0.6245 (0.0738)	0.5192 (0.1228)	0.5594 (0.1050)

From the **Table 2**, we could see that the three algorithms had their own advantages on different sub-datasets. However, it was notable that the WDD-KNN algorithm had a KNN imputation step that the other 2 missing value adaptation algorithms did not have. Generally, imputation is limited by the template dataset. Large template datasets are often owned by a few large institutions and are difficult to share for reasons such as ethical review. To their advantage, the 2 missing value adaptation algorithms can skip this step when preprocessing dataset, and the built model does not need a template dataset for prediction, which will be beneficial in practical situations.

After ensuring the reliability of our modeling results through model scores, we delved deeper into the model’s internal attributes and parameters in the following sections. This deeper analysis allowed us to extract valuable information pertinent to T2DM prediction, further validating the utility and interpretability of our algorithms.

### 3.2 Model scores provide auxiliary information other than blood glucose

**Figure 3:**
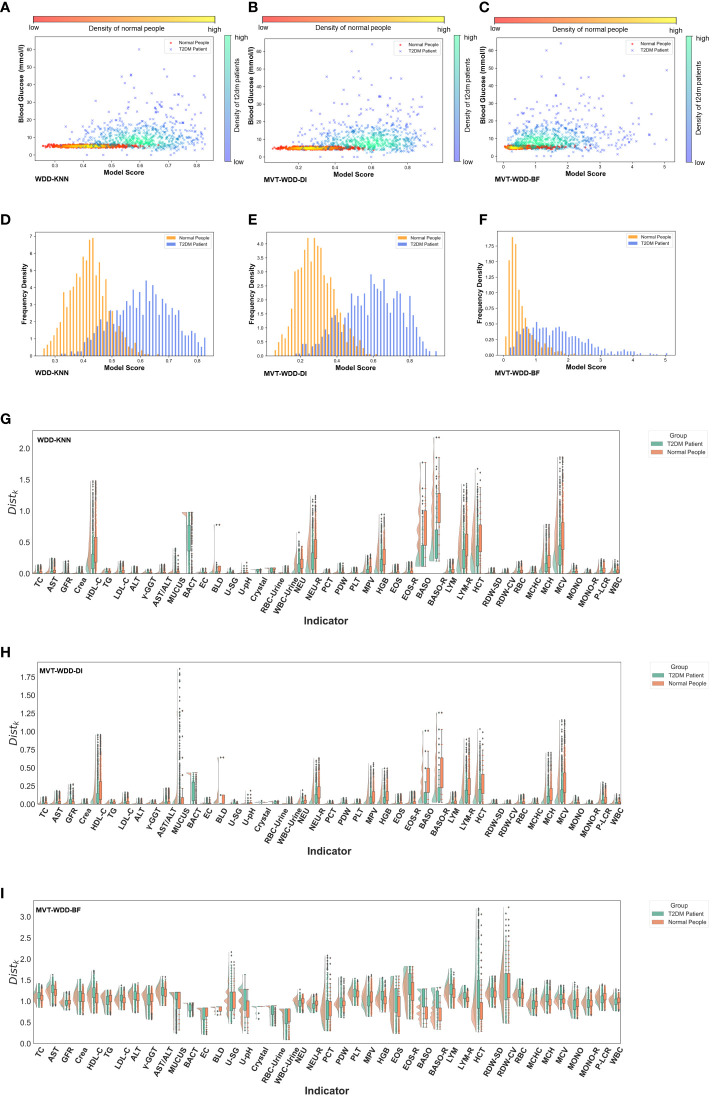
Model interpretability reflected by model scores. **(A-C)** Scatter-density heat maps of model score versus blood glucose of 3 models trained by whole PEI dataset. **(D-F)** Histograms of model score distribution of 3 models using whole PEI dataset. **(G-I)** The raincloud plots of distance scores ( $Dist_{k}$ ) of the three models using the whole PEI dataset.

The first aspect of our model’s transparency is reflected in how the distance function illustrates the model and feature contributions to predict T2DM. In physical examinations, clinicians often use blood glucose, sometimes with urine glucose as a reference, to initially assess whether a person may have T2DM. For WDD, every sample was given a risk score (Dist for each algorithm, see Method details) by the models and classified according to a risk threshold. We compared the risk stratification through blood glucose and the risk scores (Dist) from our models (**Figures 3A–C**).

With the PEI dataset (**Figures 3A–F**), we saw that in the models WDD-KNN, MVT-WDD-DI, and MVT-WDD-BF gave scores of confirmed T2DM patients that clustered in the ranges of 0.5-0.75, 0.4-0.82, and 0.4-3, respectively, while the physical examination population was clustered in the score ranges of 0.3-0.53, 0.1-0.5, and 0-1, respectively. All three models performed well in distinguishing the two populations, with WDD-KNN working best. However, it was difficult to completely distinguish the two groups if they were separated only by the level of blood glucose (**Figures 3A–C**), and many people with T2DM still had the same blood glucose levels as normal people. Also, we analysed the risk scores on the three sub-datasets in **Supplementary Note 6**.

**Figure 4:**
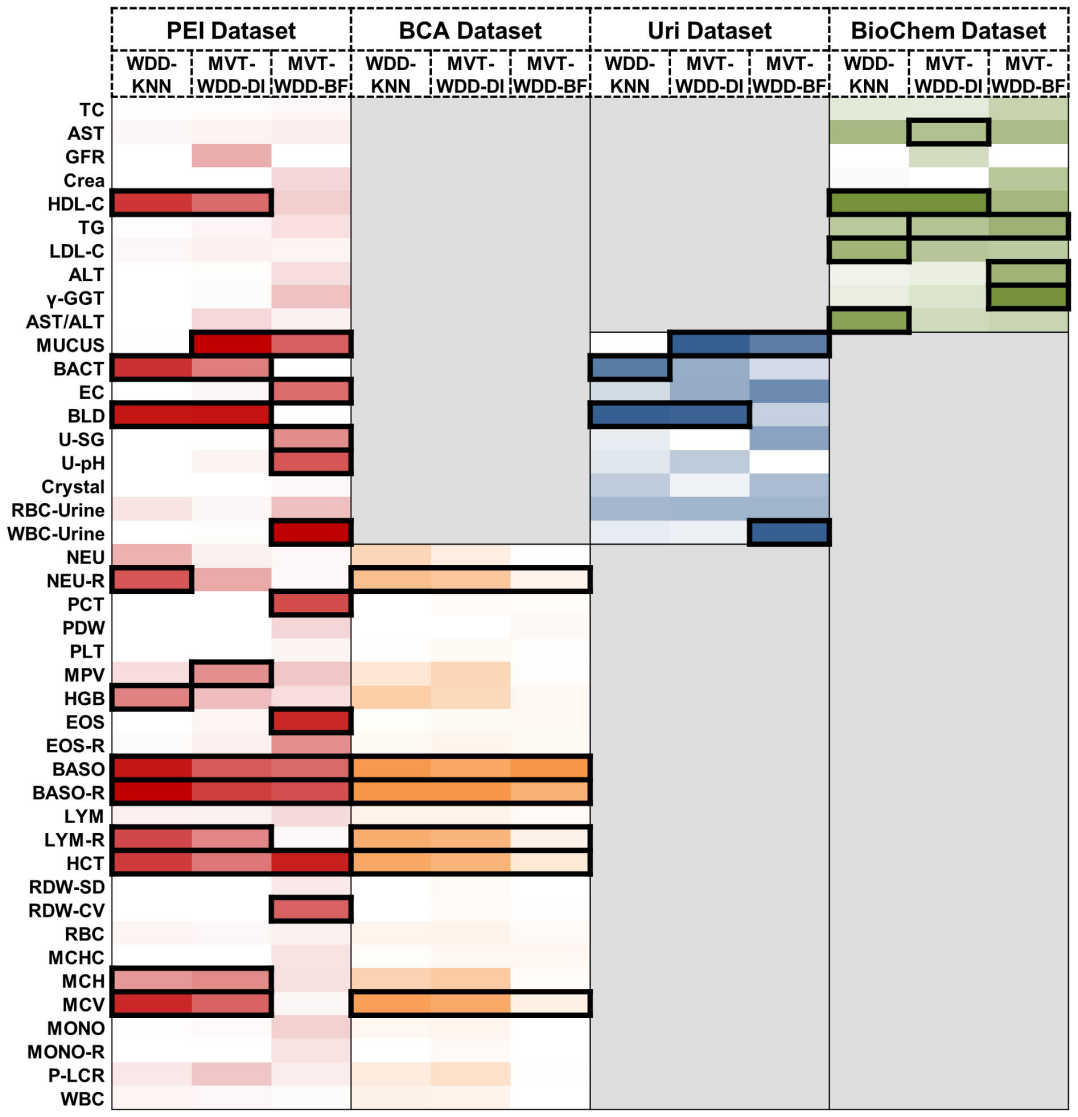
Important feature weights from different algorithms and datasets. Heat map based on the normalized feature weight values. The summation of a column (an algorithm) is 1. Darker colors represent larger weight values. The top 25% features in every column are framed by black rectangle.

To provide more information on the importance of the EHR features to every patient, we also calculated the ${\text{Dist}}_{\text{k}}$ score (see Method details) for each feature between the patients and normal people (**Figures 3G–I**; **Supplementary Figure S3**). As we can see, the selected important features mostly had different scores by each model. For example, in the model built on the PEI dataset using the MVT-WDD-DI algorithm, the ${\text{Dist}}_{\text{k}}$ scores of selected important features (**Figures 3H**, **4**) such as Bact, BLD, Baso, Baso-R, and MCV were much higher than those of other features. T2DM patients and normal people could be well distinguished by the ${\text{Dist}}_{\text{k}}$ score of these important features. The result indicates that our model assigns higher significance to features with more pronounced differences, identifying them as important and thus selecting them as effective indicators for T2DM screening.

The results show that our model effectively differentiated T2DM patients from healthy individuals in the PEI dataset, as shown in the scatter density heat map. Visualized model scores underscored performance differences, revealing potential for early T2DM detection. Notably, normal blood glucose levels don’t rule out T2DM, highlighting our model’s diagnostic value. Additionally, by analyzing the model’s distance function, we gained a deeper understanding of the mechanism behind the model’s selection of important features, enhancing our comprehension of the model.

### 3.3 The important indicators for T2DM diagnosis selected from different models

After understanding the scoring mechanism of the model and the mechanism for selecting important features, in this section and **Supplementary Notes 3** and **7**, we identified crucial diagnostic indicators for T2DM and analyzed the significance of the selected indicators for T2DM combining clinical knowledge.

The feature’s significance is determined by its weight within the models, identifying the indicators most associated with T2DM. Important features were defined as those ranking in the top 25% by weight across the 12 models. We visually represented this distribution of relative feature weights with a histogram and the specific details of these crucial features are detailed in **Figure 4**. We employed the Mann-Whitney U test to evaluate the level of feature differences between the T2DM and normal groups, finding that the selected important features exhibited significant differences (P value< 0.0001) (**Supplementary Table S6**). In addition, we compared the important features selected by using the internal weights of WDD with least absolute shrinkage and selection operator (LASSO) regression and SHAP framework. The important features showed certain consistency (**Supplementary Note 9**, **Supplementary Information Figures S14, S15**).

When the three algorithms were applied each dataset, the selected important features intersected. For example, on the PEI dataset, the indicators judged as important features by all three models were BASO, BASO-R, and HCT, and the features given high weights by two of the three models were HDL-C, MUCUS, BACT, BLD, LYM-R, MCH, and MCV. In this dataset, the AUCs of all three models were higher than 0.88, so these indicators were selected as having great significance for T2DM prediction (**Figure 4**). On the BCA dataset, the important features selected by the three algorithms are the same, which further proves the potential diagnostic value of these indicators. Moreover, we analysed the same and different important features extracted by the three algorithms, details are shown in **Supplementary Note 7**.

We conducted a literature review to integrate our clinical expertise with published research findings and investigate the clinical correlations between these important features and T2DM. Our analysis revealed that most of these important features are shown to have direct or indirect associations with T2DM. For example, urinary tract infections are known to be correlated with diabetes (28) and some indicators associated with urinary tract infections, such as haematuria and bacteria in urine, have been selected as important biomarkers. The details are collated in the **Supplementary Note 3**.

### 3.4 Multi-model analysis reveals characteristics among different age and sex groups

Leveraging our model’s transparency and feature extraction capabilities, we conducted group modeling for populations with varying demographic characteristics to unearth the diagnostic value of indicators across different groups. To mitigate the potential model bias introduced by data imbalance, we ensured basic balance in the sample volume of each age and sex category for both T2DM patients and normal individuals, as illustrated in **Supplementary Figure S5** and **Supplementary Table S2**.

**Figure 5:**
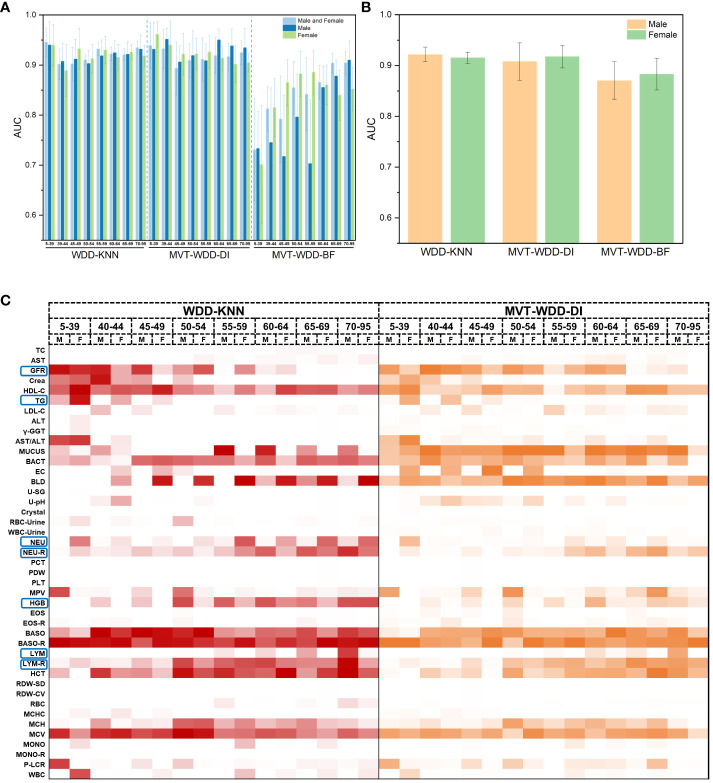
Model performance of 10-fold cross validation and feature importance in different age and sex groups. **(A)** The AUC values of the three algorithms when modeling male, female and both sexes of different ages. Error bars were generated by 10-fold cross validation (error bar represents standard deviation). **(B)** The AUC values of the three algorithms when modeling male and female of all ages. **(C)** Heat map of normalized feature weight values extracted from the model for male and female of different ages, ‘M’ represents male, ‘F’ represents female.

In most cases, age and sex will be correlated with T2DM incidence, which indicates that T2DM in different age and sex groups might have different characteristics. To further explore the importance of each feature for T2DM prediction in different age and sex groups, we tried three additional ways to divide the PEI Dataset: i. by age; ii. by sex; and iii. by age and sex. The number and proportion of people in different age and sex groups are shown in **Supplementary Figure S6** and **Supplementary Table S2**. After the division of the datasets, 26 sub-datasets (8 ages + 2 sexes + 8 ages × 2 sexes) were generated, and 78 additional models (26 datasets × 3 models) were built. The performance of each model is shown in **Figures 5A, B**. The WDD-KNN and MVT-WDD-DI algorithms performed well on each group of datasets, with AUC values mostly above 0.9, while the performance of MVT-WDD-BF was not as good. Therefore, in the subsequent analysis, only the weights of the first two algorithms were taken into consideration.

The results showed that the importance of the clinical indicators varied in different age and sex groups (**Figure 5C**; **Supplementary Figure S6**). We not only integrated the results of these models using the WDD-KNN and MVT-WDD-DI algorithms but also analysed the distribution of their measured values (**Supplementary Figures S7-13**) to explain the various importance of these indicators for T2DM diagnosis in the different groups.

**Figure 6:**
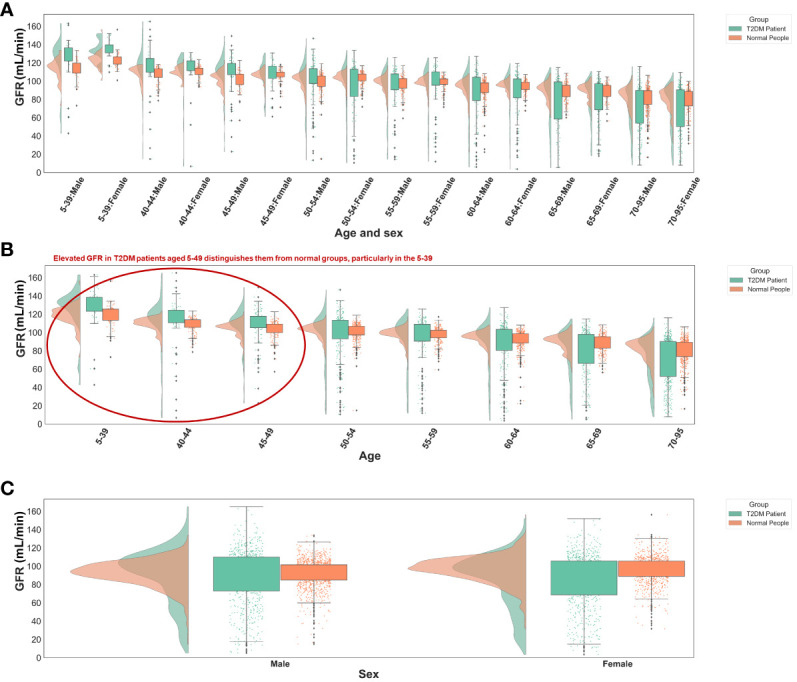
Distribution of GFR values in different groups. **(A)** Different age and sex groups. **(B)** Different age groups. **(C)** Different sex groups. All the GFR values were from origin EHRs.

The significance of glomerular filtration rate (GFR) in T2DM diagnosis diminishes with age, showing greater importance in the 5-49 age group (**Figures 5C**, **6B**). Elevated GFR in T2DM patients aged 5-49 distinguishes them from normal groups, particularly in the 5-39 (**Figure 6**). This aligns with studies linking diabetes and GFR, where early diabetic kidney disease (DKD) phases show increased GFR due to various changes in ultrastructural, vascular, and tubular factors (29). As renal health declines, GFR decreases (29, 30). Our findings suggest that GFR’s diagnostic value for T2DM varies across ages, particularly useful for early screening in younger populations, extending beyond its role in DKD.

Triglycerides (TG) showed greater significance in the 5-39 age group compared to others (**Figure 5C**; **Supplementary Figure S6**), with notable differences in TG distribution between T2DM patients and normal individuals in this age range (**Supplementary Figure S7**). This variation is attributed to age-related dietary and metabolic differences and a genetic link identified by Saxena, R. et al. (31) High TG levels in T2DM patients are associated with increased cardiovascular risks (32) and metabolic changes (33). Our model emphasizes TG’s importance in T2DM, especially in younger age groups, aligning with current research trends.

Haemoglobin (HGB) was more important in the 55- to 95-year-old group (**Supplementary Figures S5C, S6A**). In T2DM patients, as age increased, their lower HGB compared to that in normal people became more pronounced (**Supplementary Figure S12**). Based on our knowledge and experience, anaemia is diagnosed by HGB decline, so the association between anaemia and T2DM might be the use of metformin. There are reports supporting that long-term metformin use in T2DM patients can cause anaemia (34, 35), and our EHR included patients who used metformin since metformin has been a commonly prescribed drug for T2DM patients for decades. Similarly, in diabetic patients with chronic kidney disease (CKD), some factors cause iron-deficiency anaemia, such as low intestinal absorption and gastrointestinal bleeding (36). In addition, erythropoietin deficiency and hyporesponsiveness can lead to anaemia in diabetic patients with CKD (36–38). Nephrotic syndrome, characterized by oedema, hypoalbuminaemia, dyslipidaemia, and increased transferrin catabolism, contributes to anaemia due to iron and erythropoietin deficiency (36, 39, 40). Long-term administration of angiotensin-converting enzyme (ACE) inhibitors and angiotensin receptor antagonists in diabetic patients also leads to a reversible decrease in HGB through a direct blockade of the proerythropoietic effects of angiotensin II on red cell precursors, degradation of physiological inhibitors of haematopoiesis, and suppression of IGF-I (36, 41). Thus, based on many studies and reports, taking HGB as an important feature will be a useful indicator for older patients with longer duration of diabetes, so HGB was selected after modelling the EHR data. In other words, HGB decline might be a marker of T2DM or T2DM-correlated disease, but it might be interfered with by some confounding factors, so its use for early diagnosis might be limited. This limitation is caused by the lack of medication information in our EHR data. Despite our meticulous selection of the patient’s first record within the hospital system, we cannot guarantee that they have not undergone therapeutic interventions at other institutions. To address this shortcoming, cohort studies with long-term follow-up are needed.

The Neutrophils (NEU), neutrophil rate (NEU-R), lymphocyte rate (LYM), and lymphocyte rate (LYM-R) have also been observed to correlate with age or sex, as discussed in **Supplementary Note 8**.

The result of this section demonstrates that through group modeling and the model’s feature extraction capability, we identified several age and sex-related biomarkers for T2DM prediction. Integrating insights from the results, it’s evident that our model can glean valuable auxiliary diagnostic information from internal parameters like feature weights and distance functions. This effectively showcases the algorithm’s transparency throughout the modeling process, highlighting its capacity to provide interpretable insights crucial for clinical application.

## 4 Discussion

Here, we developed WDD framework-based models for the prediction of T2DM using physical examination features in EHRs. Using the model parameters, the importance of the features can be measured by the distance between the sample and optimal point. Based on our investigation, the top 25% of indicators were found to be directly or indirectly related to T2DM, offering potential value as diagnostic markers for T2DM.

In our analysis, a variety of white blood cells—neutrophils, basophils, eosinophils, and lymphocytes—emerged as significant features. This aligns with existing literature, which indicates that T2DM patients experiencing concurrent infections may exhibit inflammation, leading to an altered white blood cell count (42, 43). While current research posits that the count or ratio levels of these white cells alone do not suffice as diabetes risk factors, a multifaceted approach is often necessary. For instance, the neutrophil-lymphocyte ratio is recognized as an independent predictor of T2DM (44). The inclusion of these white blood cells as important indicators by our model is consistent with current research insights, further affirming the potential of combining these white blood cell levels for aiding T2DM diagnosis. Moreover, this demonstrates our model’s proficiency in capturing the complex interrelationships among indicators, highlighting its diagnostic relevance.

Indicators related to red blood cells and platelets, such as mean platelet volume, plateletcrit, hematocrit, coefficient of variation of red cell distribution width, mean corpuscular volume, and mean corpuscular haemoglobin, were also identified as significant by our model. In T2DM patients experiencing insulin resistance and metabolic syndrome, the adverse metabolic conditions—including hyperglycemia, hypertension, dyslipidemia, inflammation, and impaired fibrinolysis—elevate the risk of atherosclerosis and lead to microvascular complications like diabetic retinopathy, nephropathy, and neuropathy (45, 46). Additionally, atherosclerosis, which may result from increased platelet adhesion and hypercoagulability in T2DM patients, is a key pathological mechanism behind macrovascular complications (46). These vascular complications can cause abnormalities in red blood cells and platelets. Therefore, the aforementioned indicators are linked to the common microvascular and macrovascular complications in diabetics, suggesting their potential as diagnostic markers for T2DM.

Urinalysis-related indicators, such as haematuria, leukocytes in urine, mucinous filaments, bacteria in urine, epithelial cells in urine, urine pH, and specific gravity, have been selected as significant markers by our model. These indicators are primarily associated with conditions prevalent among individuals with diabetes, such as urinary tract infections (28), which are notably common and can lead to haematuria or abnormal quantities of cells and bacteria in the urine. Moreover, the inflammation caused by these infections may result in an abnormal number of white blood cells, further validating the model’s ability to discern potential relationships between indicators. The combination of increased net acid excretion and reduced use of ammonia buffers in individuals with diabetes leads to lower urine pH (47, 48). A lower urine pH heightens the risk of nephrolithiasis, including uric acid stones (47, 49). Diabetic nephropathy may manifest through abnormal urine specific gravity, where a lower-than-normal urinary specific gravity, along with increased polyuria, signals diabetes insipidus (50). These findings underscore the interconnectivity of urinary markers with diabetes-related infections and complications, emphasizing their potential diagnostic relevance.

While individual physical examination indicators often cannot serve as standalone diagnostic criteria for T2DM, our model successfully integrates multiple indicators to construct a diagnostic model for T2DM. Leveraging the model’s high interpretability, we can determine the importance of each indicator in the diagnosis, enhancing its capability to aid in the auxiliary diagnosis of T2DM. This approach not only harnesses the collective diagnostic potential of various indicators but also provides valuable insights into their diagnostic significance, offering a refined perspective on T2DM diagnosis.

Besides, based on our clinical knowledge, the diagnosis of T2DM often correlates with demographic factors. Therefore, we segmented the data by age and gender, utilizing the feature weights provided by our model. Through this process, combined with a literature search, we identified several biomarkers related to age or sex, such as glomerular filtration rate, triglycerides, and haemoglobin. This further validates our model’s efficacy in extracting medically valuable information and illustrates that different indicators may require attention when diagnosing diabetes in patients of varying ages and sexes. This approach not only enriches the diagnostic model with nuanced clinical insights but also underscores the importance of personalized medicine in the management and treatment of T2DM.

The results show that our algorithm boasts a high degree of internal interpretability, enabling the extraction of key indicators for T2DM diagnosis without the need for third-party tools. Furthermore, by analyzing the model’s parameters (**Figure 3**), we can comprehend the mechanism behind the selection of important indicators, thereby providing reliable auxiliary diagnostic information. Additionally, our model possesses a distinct advantage as mentioned in the Methods section: benefiting from the transparency of WDD, its internal distance function is easily modifiable. Instead of imputing missing values, our approach involves ‘tolerating’ them by incorporating penalty terms into two of the algorithms. This strategy diminishes the necessity for exhaustive searches for high-quality template data during the imputation process, proving WDD to be a highly transparent and interpretable auxiliary diagnostic algorithm.

This work suggests that machine learning could extend beyond predictive accuracy to include interpretative insights, which might be useful in clinical settings. Such insights have the potential to aid clinicians in understanding the basis of diagnostic suggestions given by the model. This could lead to a more cooperative relationship between machine learning and healthcare professionals. However, the integration of these technologies in clinical practice requires careful consideration and ongoing evaluation.

## 5 Conclusion

Overall, we developed three WDD-based interpretability algorithms and built T2DM diagnostic models, identifying several relevant diagnostic indicators with potential utility in assisting T2DM diagnosis. However, it is crucial to acknowledge that the mechanisms of interaction among these indicators, as well as their causal connections with T2DM, cannot be directly deduced from the current model information. In our future work, leveraging the transparency of WDD, we plan to incorporate knowledge of causal probabilities to enhance our model further, uncover the complex relationships between indicators and T2DM.

## Data availability statement

The code used for modelling in this work is available at: https://github.com/Lvxiang713/WDD_T2DMprediction. Rearchers should contact the corresponding authors for approval to obtain and use the source data. The original contributions presented in the study are included in the article/**Supplementary Material**. Further inquiries can be directed to the corresponding authors.

## Ethics statement

The studies involving humans were approved by This study protocol was approved by the ethics committee of the Affiliated Hospital of Southwest Medical University, China (KY2022266) and the Chinese Clinical Trial Registry (ChiCTR2200064435). The studies were conducted in accordance with the local legislation and institutional requirements. Written informed consent for participation was not required from the participants or the participants' legal guardians/next of kin in accordance with the national legislation and institutional requirements.

## Author contributions

XL: Writing – review & editing, Writing – original draft, Visualization, Methodology, Formal analysis. JL: Writing – original draft, Investigation, Funding acquisition, Formal analysis, Data curation. HW: Writing – review & editing, Investigation, Data curation. HG: Writing – review & editing, Visualization, Formal analysis. XB: Writing – original draft, Investigation, Formal analysis, Data curation. PY: Writing – original draft, Data curation. ZJ: Writing – review & editing, Data curation. YZ: Writing – original draft, Investigation, Formal analysis. RJ: Writing – review & editing, Writing – original draft, Validation, Methodology, Funding acquisition, Data curation, Conceptualization. QC: Writing – original draft, Supervision, Funding acquisition, Data curation, Conceptualization. ML: Writing – review & editing, Supervision, Funding acquisition, Conceptualization.
