# Supplementary material for: Identifying diagnostic indicators for type 2 diabetes mellitus from physical examination using interpretable machine learning approach
Source: Front Endocrinol (Lausanne). 2024 Mar 18;15:1376220. doi: 10.3389/fendo.2024.1376220 (PMC10982324; doi:10.3389/fendo.2024.1376220)
Supplement: Supplementary file 1 [file DataSheet_1.docx]

Supplemental information for

**Identifying diagnostic indicators for type 2 diabetes mellitus from physical examination using machine learning approach**

**This file includes:**

Supplementary Note 1-10

Figure S1-15

Table S1-7

Supplementary Reference

Supplementary Note 1: Construction and evaluation of the prediction model

As described above, the risk prediction task in this work was a binary classification problem. An output after inference assigned the value 1 represents high risk (positive), and an output assigned 0 represents low risk (negative).

To make the prediction more reliable, we used 10-fold cross-validation and an independent test to evaluate the stability of the test. The dataset was split into a cross-validation dataset and an independent dataset at a ratio of 8:2. Additionally, we repeated the cross-validation and independent test 1000 times to reduce the influence of coincidence. Notably, the independent test dataset was consistent in the repetitions, but the splitting for cross-validation changed in every repetition.

Supplementary Note 2: Details of the training

In this work, we developed three algorithms containing different hyperparameters for optimization. We optimized $\gamma$ of WDD-KNN, $\gamma$ and $\delta$ of MVT-WDD-DI, and $\gamma$ and $\lambda$ of MVT-WDD-BF. As shown in Table S5, we initially sampled all the hyperparameters in the range $\left[ 2^{-10},2^{10} \right]$ with every integer index number. In total, WDD-KNN had 21 hyperparameters, while the other two algorithms had 441 (i.e., 21^2^) hyperparameter pairs for tuning.

Supplementary Note 3: Clinical association of important features with T2DM

We take the union of the important features extracted from the 8 models whose AUC above 0.75. Totally, 20 high-weight features were filtered out by co-occurrence (Table S5). To verify whether the information learned by our model is related to T2DM, we conducted a literature collection and combine our clinical knowledge to explore how the 20 important features clinically correlated with T2DM. To explain these features more systematically, we classified them into three categories based on our clinical medicine experience.

The first category is the indicators of blood cell. We divided them into two sub-categories according to their medical significance in association with T2DM:

I-1: Indicators of white blood cells

White blood cell is usually divided into 5 main types: neutrophils, eosinophils, basophils, lymphocytes and monocytes, all of which are included in our modeling features. Based on our analysis of feature importance, neutrophils, basophils, eosinophils and lymphocytes were important to predict T2DM (Table S5). For T2DM patients, diabetes combined with infection will bring inflammation to the body, which will induce the abnormal number of white blood cells.(1,2) Regarding to the selected sub-types, it has been reported in relevant literature that neutrophils- lymphocytes ratio can be regarded as an independent predictor of T2DM via multinomial logistic regression analysis.(3) Based on our literature research, currently there isn’t a report of the significant relation between basophil and T2DM patients, but few experimental studies in rats reported the correlation between basophils and pre-diabetes to type 2 diabetes.(4)

I-2: Indicators of red blood cells (RBCs) and platelets

In this work, six indicators relating to RBCs and platelets were selected as the important features correlating to T2DM, including mean platelet volume, plateletcrit, haematocrit, coefficient of variation of red cell distribution width, mean corpuscular volume and mean corpuscular haemoglobin (Table S5). For T2DM patients with insulin resistance and metabolic syndrome, the toxic metabolic environment including hyperglycemia, hypertension, dyslipidemia, inflammation and impaired fibrinolysis will increases the atherosclerotic risk and causes microvascular complications like diabetic retinopathy, nephropathy, neuropathy.(5,6) Besides, the process of atherosclerosis is the pathological mechanism of macrovascular which may be caused by the increased platelet adhesion, hypercoagulability of T2DM patients.(6)These vascular complications will lead to the abnormalities of red blood cells and platelets. More specifically, mean platelet volume is a useful biomarker for cardiovascular disease according to Chu, S. G., et al.’s meta-analysis, the mechanisms of action of mean platelet volume may be related to the different enzyme activity of the larger and smaller platelets(7) and plateletcrit is statistically related to the diabetic neuropathy.(8) Haematocrit is independently associated with cardiovascular events in T2DM patients because low haematocrit may predispose to chronic myocardial hypoxia left ventricular dilatation and dysfunction.(9) Coefficient of variation of red cell distribution width associated with diabetic nephropathy, mean corpuscular volume associated with chronic kidney disease and mean corpuscular haemoglobin associated with diabetic retinopathy were obtained by statistical analysis.(10–12)

The second category contains ten clinical indicators in physical examination biochemical items, in which only high-density lipoprotein cholesterol (HDL-C) was selected as the important feature (Table S5). HDL-C is related to blood lipids and one common type of cholesterol in the human body. High density lipoprotein cholesterol can transport blood lipids in blood vessels to the liver for processing, as a protective factor of cardiovascular disease. Recent studies have shown low mean and high variability in HDL-C were independent predictors of diabetes with an additive effect.(13)

The last category consists of nine indicators relevant to routine urine items. Totally seven of them were selected as the important feature. According to the medical significance in association with T2DM, we divided the features into three sub-categories for discussion:

III-1: Indicators of urinary tract infections

Haematuria, leukocytes in urine, mucinous filament, bacteria in urine and epithelial cells in urine were selected as the important features according to the feature weights (Table S5), and all the six features were relevant to urinary tract infections. Urinary tract infections are one of the most common types of infections in people with diabetes,(14) which can lead to haematuria or abnormal numbers of cells and bacteria in the urine. All of these indicators reflect the severity of inflammation caused by urinary tract infections in diabetic people. The appearance of these inflammations can also lead to an abnormal number of white blood cells in I-1.

III-2: Urine pH and specific gravity

These two indicators are both physical and chemical properties of urine, where specific gravity refers to the ratio of the weight of urine to the same volume of pure water. In diabetics patients, it has been reported that the combination of greater net acid excretion and lower use of ammonia buffers will lead to a lower urine pH.(15,16) Also, lower urine pH increases the risk for nephrolithiasis such as uric acid stones.(15,17) Diabetics nephropathy may be accompanied by abnormal urine specific gravity. The lower normal urinary specific gravity accompanied by worsening polyuria is a signal for diabetes insipidus.(18)

In summary, we have further verified the important features extracted in combination with clinical knowledge, and classified and summarized them, which reflects what our models have learned from the EHRs data. We have divided these features into a total of three categories according to their clinical significance. These categories of indicators deserve special attention in predicting, as well as early screening for T2DM. From another hand, it could be proved that our model has the ability to extract features relevant to the target task by analyzing the association between these important features and T2DM.

Supplementary Note 4: Biased distribution misleading model using features

In the process of modeling with the MVT-WDD-DI algorithm, we did not balance the missing rate of features of positive and negative samples in the initial version. When the MVT-WDD-DI algorithm was used to model PEI Dataset and Uri Dataset, the AUC values were as high as 0.9836 and 0.9796, respectively. In addition, MVT-WDD-DI only selected BACT as important features in these two datasets. Therefore, we went back to the original data set to calculate the deletion rate of BACT and found that the deletion rate of this feature in T2DM people was as high as 49.31%, while the deletion rate of this feature in Normal population was only 0.4%. It is obviously unreasonable for the model to still have such a good performance. Then we analyzed the $\mathrm{Dis}t_{k}$ of each feature and found that the $\mathrm{Dis}t_{k}$ of BACT was much higher than other features in the above model (Figure S13). Since the $\mathrm{Dis}t_{k}$ will be summed in the process of sample score calculation by MVT-WDD-DI, the model basically uses BACT as a criterion to classify the two groups of people. Coincidentally, this algorithm assigns high $\mathrm{Dis}t_{k}$ to normal people and low $\mathrm{Dis}t_{k}$ to T2DM People during modeling and MVT-WDD-DI will set the missing value as 0, which directly leads to the model judging the samples missing BACT as T2DM people. This further leads to inflated results.

Therefore, we balanced the deletion rate of each feature of the positive and negative samples, and the specific steps are as follows: (1) The deletion rate of each feature in positive and negative samples $DeRaP_{k}, DeRaN_{k}$ were calculated respectively. (2) $maxDeRa_{k}=max(DeRaP_{k},DeRaN_{k})$ . (3) The features of positive and negative samples are randomly masked as missing value to ensure that the missing rate of the kth-feature in the positive and negative samples is $maxDeRa_{k}$.

Supplementary Note 5: Details of the training step

We implemented all the algorithms using the PyTorch library. We chose the full batch as the updating scheme. After a few initial attempts, we limited the parameters of $s_{k}$ to being updated at the first 5 of every 30 epochs, while other parameters were updated every epoch. The maximum number of epochs was set as 50000, and an early stop mechanism was employed to end the training process when the difference between the last two epochs of the loss value was less than 10^-6^. The learning rate was set to 0.001 when the parameter of $s_{k}$ was updated and to 10^-2^ otherwise.

An initial step was executed before training. In this step, all the initial parameters of $s_{k}$ were set to $\frac{1}{f}$ for $f$ features, while the initial parameters of $\boldsymbol{x}$ were randomly generated from a uniform distribution in the range $\left[ \boldsymbol{\mu}_{\boldsymbol{B}^{+}}-2\boldsymbol{\sigma}_{\boldsymbol{B}^{+}},\boldsymbol{\mu}_{\boldsymbol{B}^{+}}+2\boldsymbol{\sigma}_{\boldsymbol{B}^{+}} \right]$, where $\boldsymbol{\mu}_{\boldsymbol{B}^{+}}$ and $\boldsymbol{\sigma}_{\boldsymbol{B}^{+}}$ were the average and variance of all the instances in positive samples. The initial step of $\boldsymbol{x}$ was repeated until the loss function received a gradient in the first backwards step. Using the range $\left[ \boldsymbol{\mu}_{\boldsymbol{B}^{+}}-2\boldsymbol{\sigma}_{\boldsymbol{B}^{+}},\boldsymbol{\mu}_{\boldsymbol{B}^{+}}+2\boldsymbol{\sigma}_{\boldsymbol{B}^{+}} \right]$ as the sampling space, point x should be as close to positive samples and as far from the negative samples as possible; thus, the initial position should not be far from the positive samples.

Supplementary Note 6: The risk scores on the three sub-datasets

In the three sub-datasets (Figure S1, 2), it is intuitively seen from the graph that the MVT-WDD-DI algorithm had the best effect on distinguishing between the two groups of people by the model scores in the Uri Dataset. WDD-KNN behaved similarly on the Uri Dataset and BCA Dataset. The MVT-WDD-BF model performed worse than the others in the Uri dataset, with the transverse axis centres of the two groups almost coinciding (Figure S1f, 2f). On the BioChem Dataset, regardless of which algorithm was used, the classification performance of the score was relatively weak (Figure S1g-i, 2g-i)

Supplementary Note 7: The important features selected by the different algorithms

The feature weight values of MVT-WDD-BF differ from those of two other algorithms (Figure 4), which supports the idea that the models may process the features in different ways. From an algorithmic point of view, this is because MVT-WDD-BF uses a different distance function than the other two algorithms (see Algorithm). However, MVT-WDD-BF also had 3 and 4 overlapping important features with the other two models, respectively, while WDD-KNN and MVT-WDD-DI selected 9 of the same important features on the PEI dataset (Figure 4, Figure S4). In addition, three algorithms selected the same important features on BCA datasets (Figure 4). These findings indicate that although the algorithms have different underlying principles, they still extract overlapping information.

Supplementary Note 8: Diagnostic indicators related to age or sex

The importance of neutrophils (NEU), neutrophil rate (NEU-R), lymphocytes (LYM) and lymphocyte rate (LYM-R) increased with age (Figure 5c, Figure S6a). NEU was more important among females than males, while LYM was the opposite (Figure S6b). In fact, the distribution of these four indicators was progressively more differentiated between the two T2DM/normal groups as age increased (Figure S8b, 9b, 10b, 11b). Based on our literature search, few studies have reported the association of T2DM with these separate indicators, but various studies have reported the association of T2DM with their ratio, the neutrophil/lymphocyte ratio (NLR). An elevated NLR is positively correlated with the incidence of T2DM(3,19), and the NLR is positively correlated with the degree of glucose intolerance and insulin resistance, according to the Chennai Urban Rural Epidemiology Study.(20) Verma, S. et al. showed that the NLR predicted adverse cardiovascular events in patients with T2DM, and the higher NLR tercile was overrepresented by the older age group or in those with a longer disease duration of diabetes,(21) which is consistent with our finding that these four features are more important for T2DM diagnosis at higher ages. The NLR is an adjunctive prognostic indicator of cardiovascular complications for patients with glucose intolerance(20). As a reflection of this, our models selected all four of the above indicators as important features, which could be evidence supporting the importance of the NLR. Importantly, all four indicators can be obtained from routine blood cell analysis. Considering that both NEU and LYM show age-related correlations, we believe that our models could find the features with simple linear correlations and can shrink the search space to fewer age groups.

Supplementary Note 9: Compared with LASSO and SHAP

We explored the use of the LASSO algorithm and a combination of Random Forest with Shapley Additive explanations (SHAP) for important feature selection. For the LASSO algorithm, we followed the approach described in previous work(22), employing a nested tenfold cross-validation (with the inner loop determining the regularization parameter, λ) over 100 bootstrap samples (outer loop) drawn from the feature selection set. Each clinical indicator was assigned a score between 0 to 100, and an indicator was defined as an important feature if it was selected in over 95% of the bootstrap samples (Supplementary information Figure S14). For feature selection using the combination of Random Forest and SHAP, we adhered to the original methodology, selecting the top 25% of features based on SHAP values (Supplementary information Figure S15). The important features identified by either the LASSO or SHAP showed considerable consistency with those selected through model parameters using the WDD method. For instance, when comparing the top 25% of important features identified by the WDD-KNN algorithm with those selected by the SHAP algorithm on the PEI dataset, the intersection consisted of 9 out of 11 features. Similarly, for the MVT-WDD-DI algorithm compared to the SHAP algorithm, the intersection included 8 out of 11 features. This further validates the reliability of our method in selecting important features.

Supplementary Note 10: Modeling with BMI

Body Mass Index (BMI) has been reported to be associated with T2DM prediction(23), and we tried to include BMI for modeling. It is observed that the inclusion of the BMI had a minimal impact on the WDD-KNN and MVT-WDD-DI algorithms, with the average fluctuation in repeated AUC values decreasing and increasing by approximately 0.001, respectively. For the MVT-WDD-BF algorithm, the improvement was only about 0.01. Overall, adding BMI did not significantly affect the model outcomes.

This minimal impact may be attributed to the fact that our PEI dataset already includes indicators related to BMI, such as HDL-C and TG, which have been reported in the literature(24).

Supplementary Figure


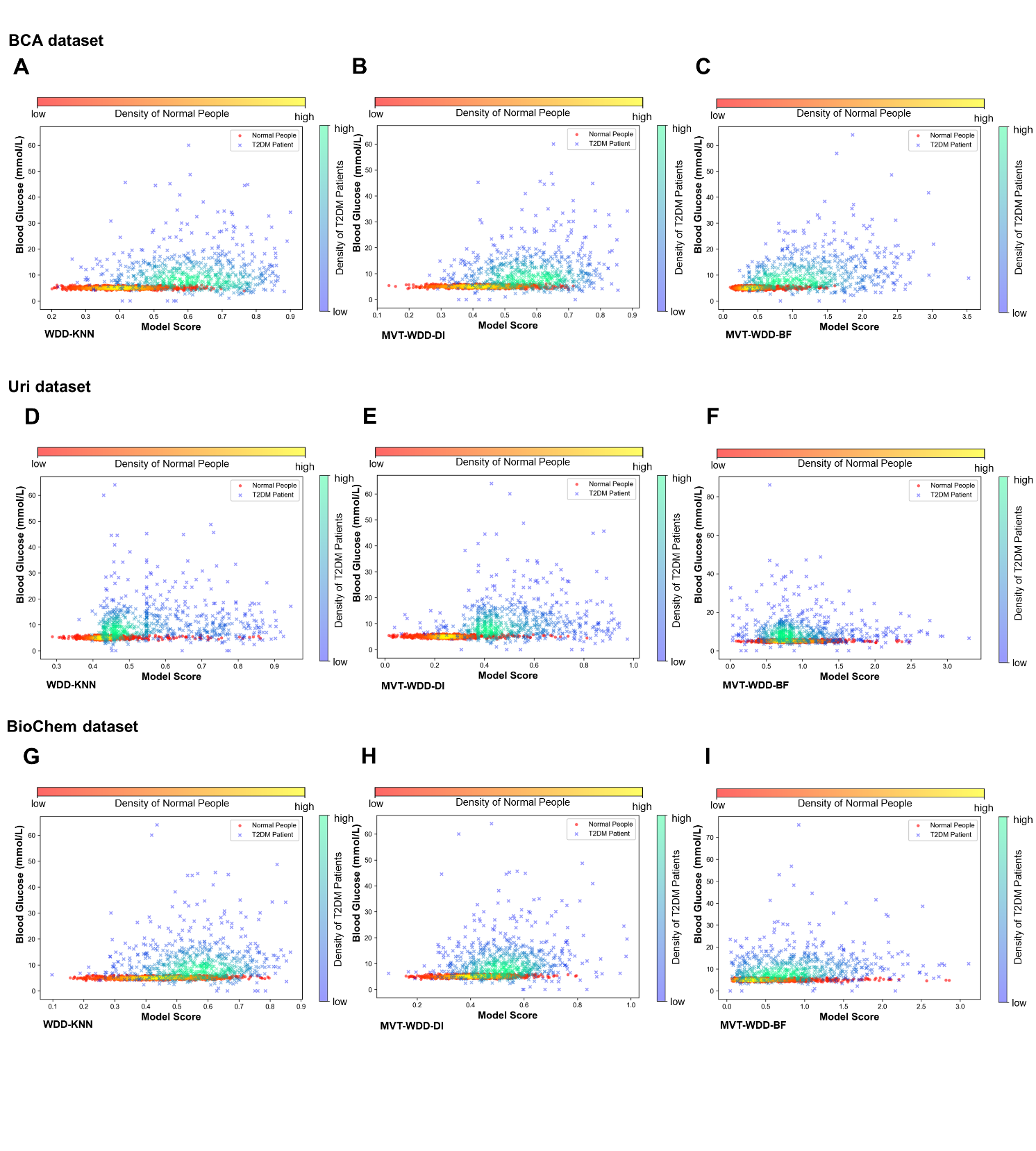


**Figure S1. Scatter-density heat maps of model score versus blood glucose. (A**-C) Three models trained by BCA dataset. **(D-F)** Three models trained by Uri dataset. **(G-I)** Three models trained by BioChem dataset.

**
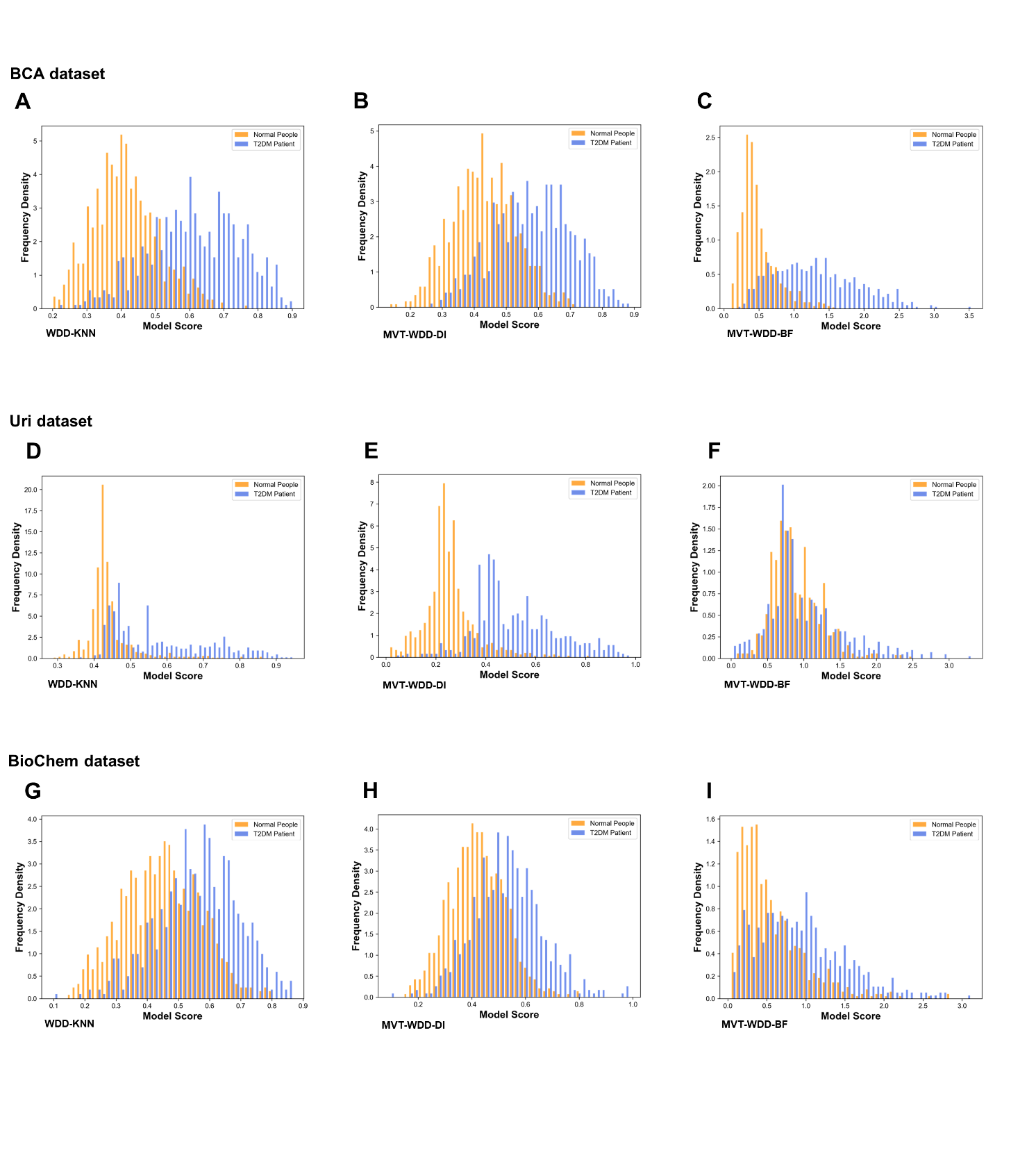
**

**Figure S2. Histogram of model score distribution for comparing the normal people and T2DM patients. (A-C)** The distributions of three models trained by BCA dataset. **(D-F)** The distributions of three models trained by Uri dataset. (**G-I)** The distributions of three models trained by BioChem dataset.


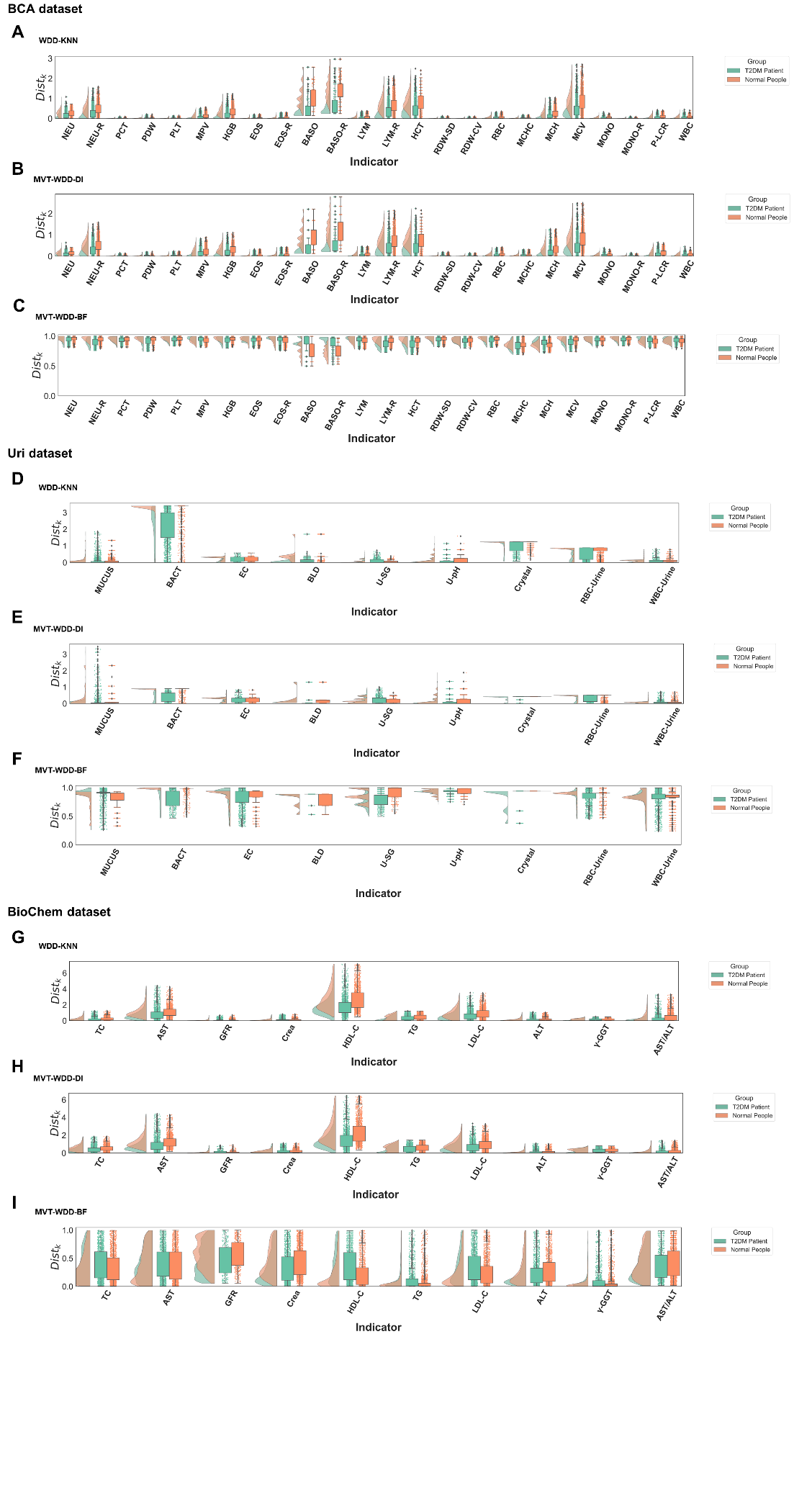


**Figure S3. The Raincloud plots of distance scores (**$\boldsymbol{Dis}\boldsymbol{t}_{\boldsymbol{k}}$**) for comparing the normal people and T2DM patients. (A-C)** The plots of three models trained by BCA dataset. **(D-F)** The plots of three models trained by Uri dataset. **(G-I)** The plots of three models trained by BioChem dataset.


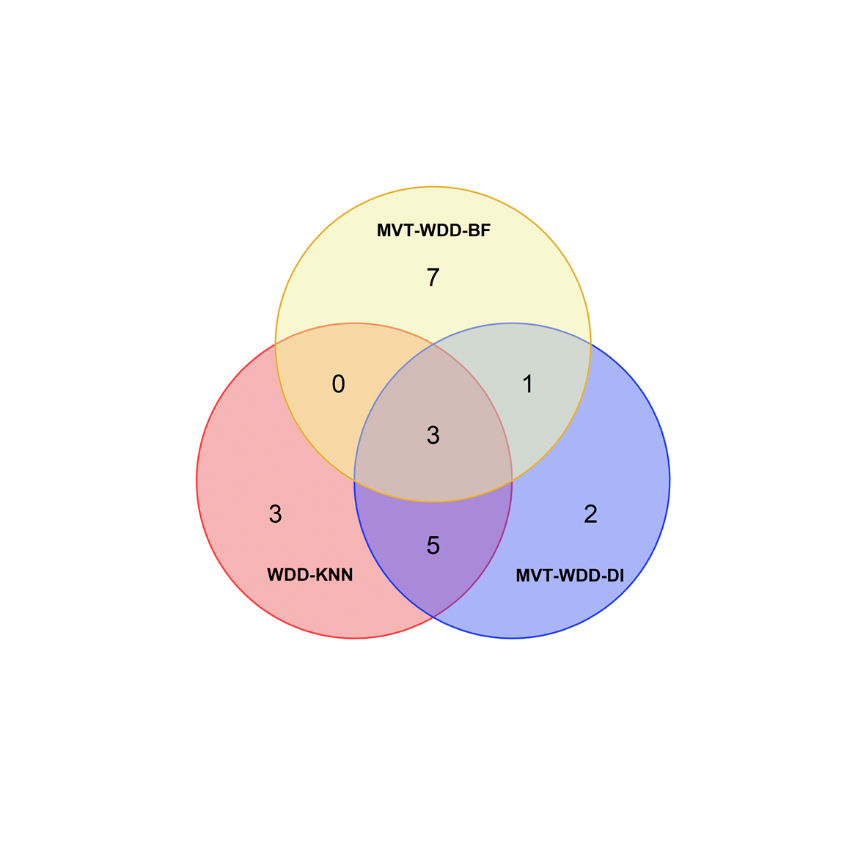


**Figure S4. Venn diagram of** **important features extracted from the three models using PEI dataset.**


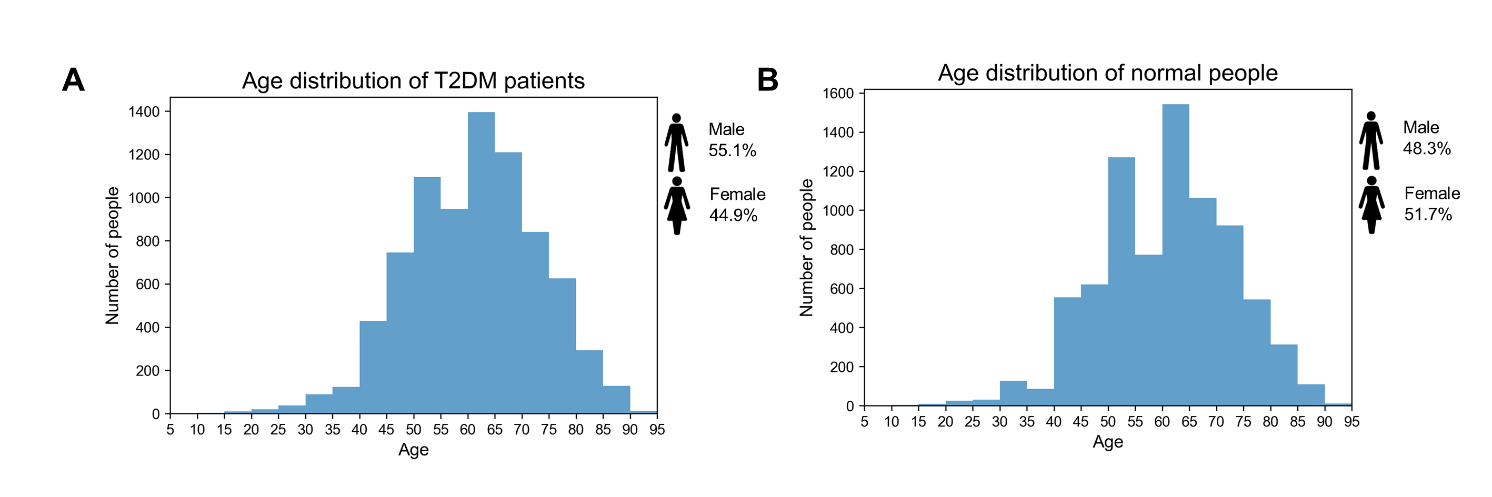


**Figure S5. Age and sex distribution of normal people and T2DM patients.** The age distribution of the two groups of people was calculated in an interval of five years.


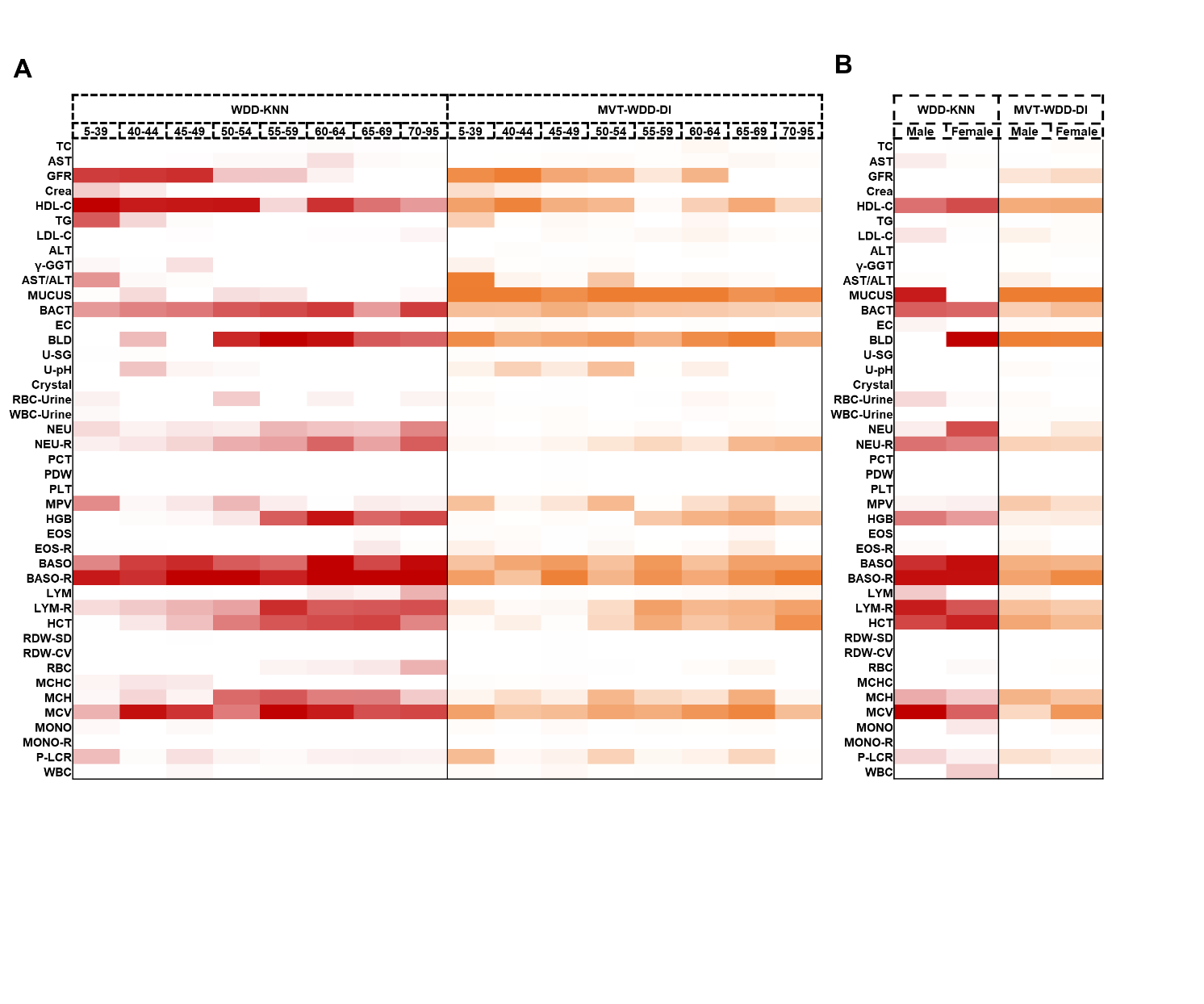


**Figure S6. Heat map of normalized feature weight values** a Different age groups b Different sex groups. In every column (i.e. age or sex group), the summation is 1.


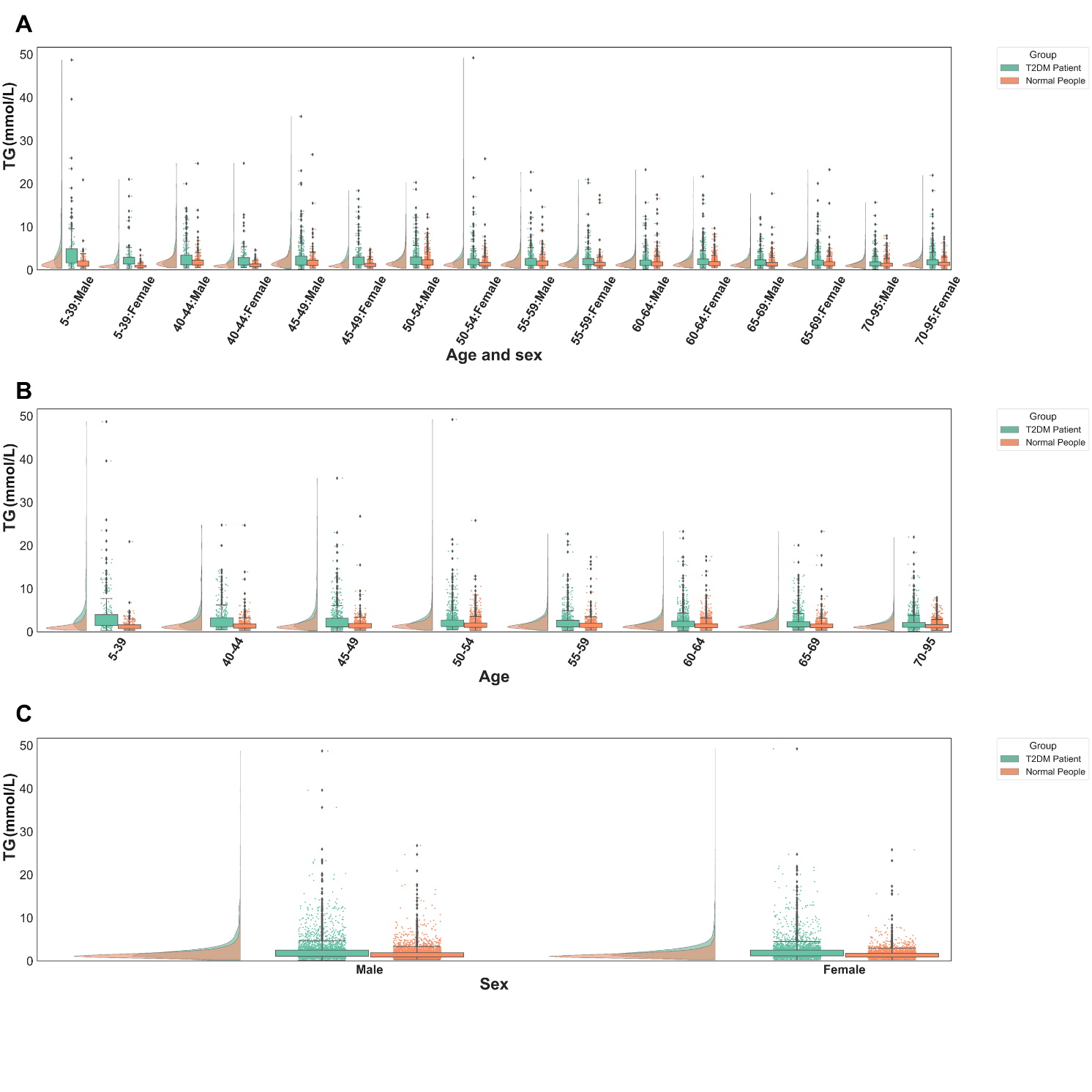


**Figure S7. Distribution of measured TG values in different age and sex groups. (A)** Different age and sex groups. **(B)** Different age groups. **(C)** Different sex groups. All the TG values were from origin EHR**.**

**
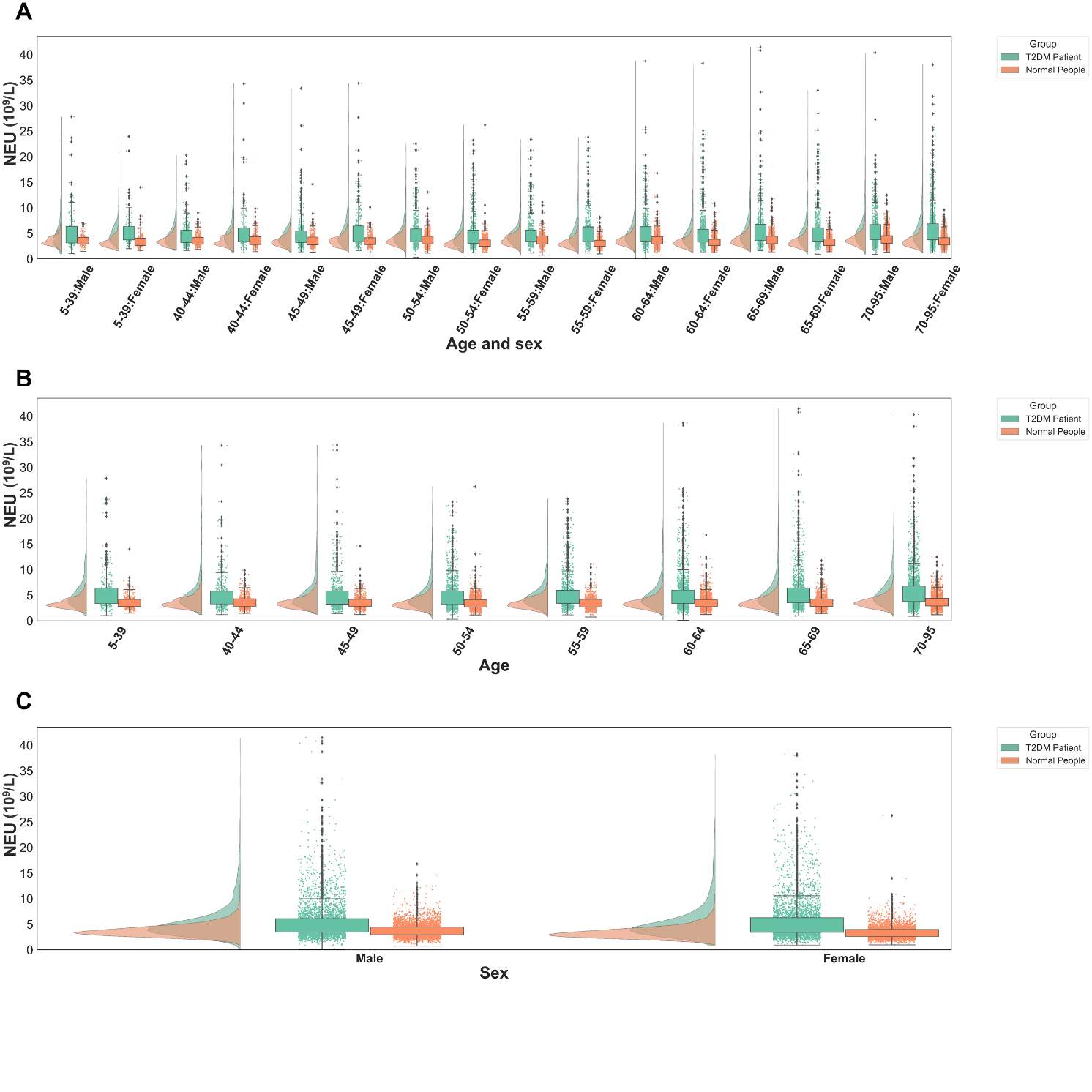
**

**Figure S8. Distribution of measured NEU values in different age and sex groups. (A)** Different age and sex groups. **(B)** Different age groups. **(C)** Different sex groups. All the NEU values were from origin EHR**.**

**
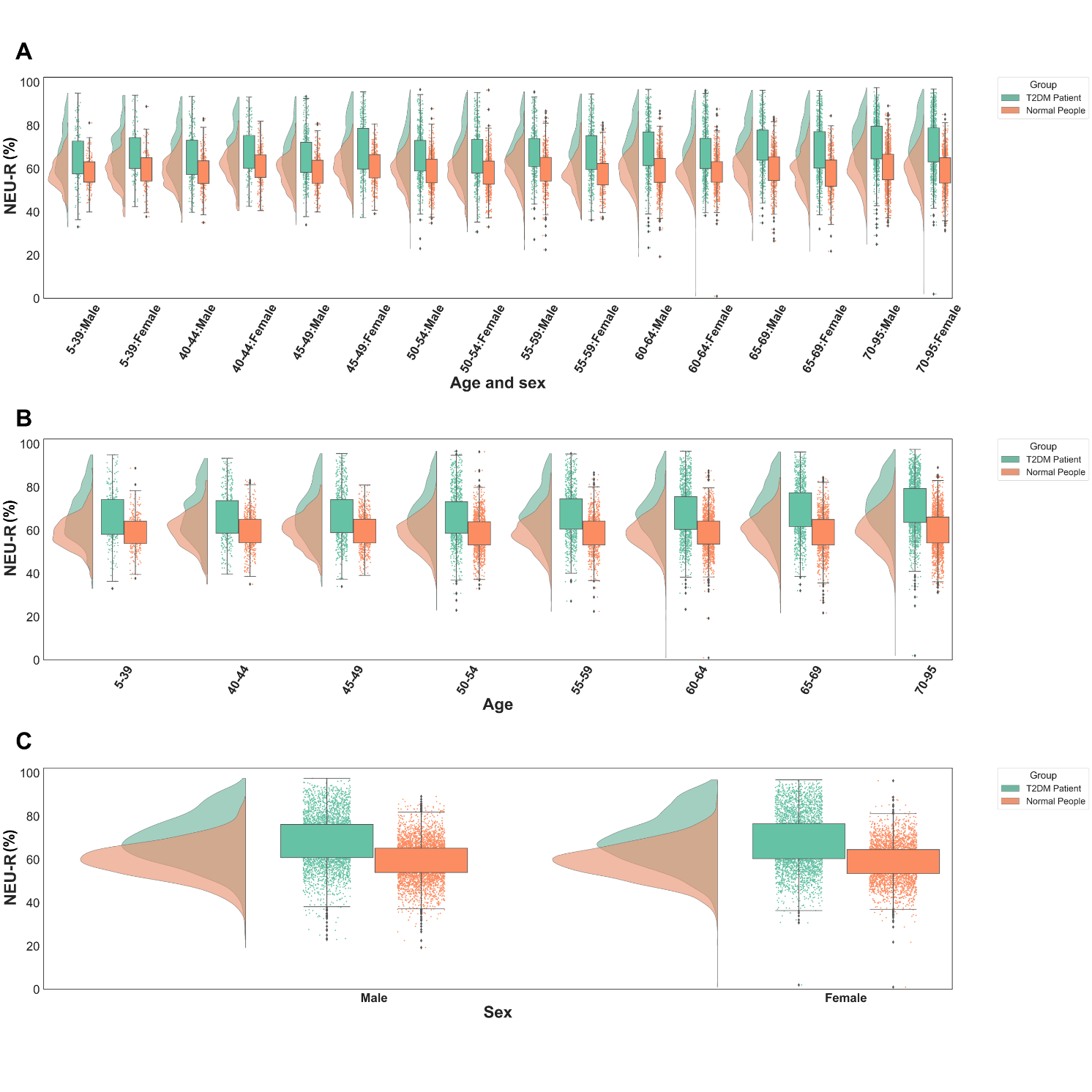
**

**Figure S9. Distribution of measured NEU-R values in different age and sex groups. (A)** Different age and sex groups. **(B)** Different age groups. **(C)** Different sex groups. All the NEU-R values were from origin EHR**.**

**
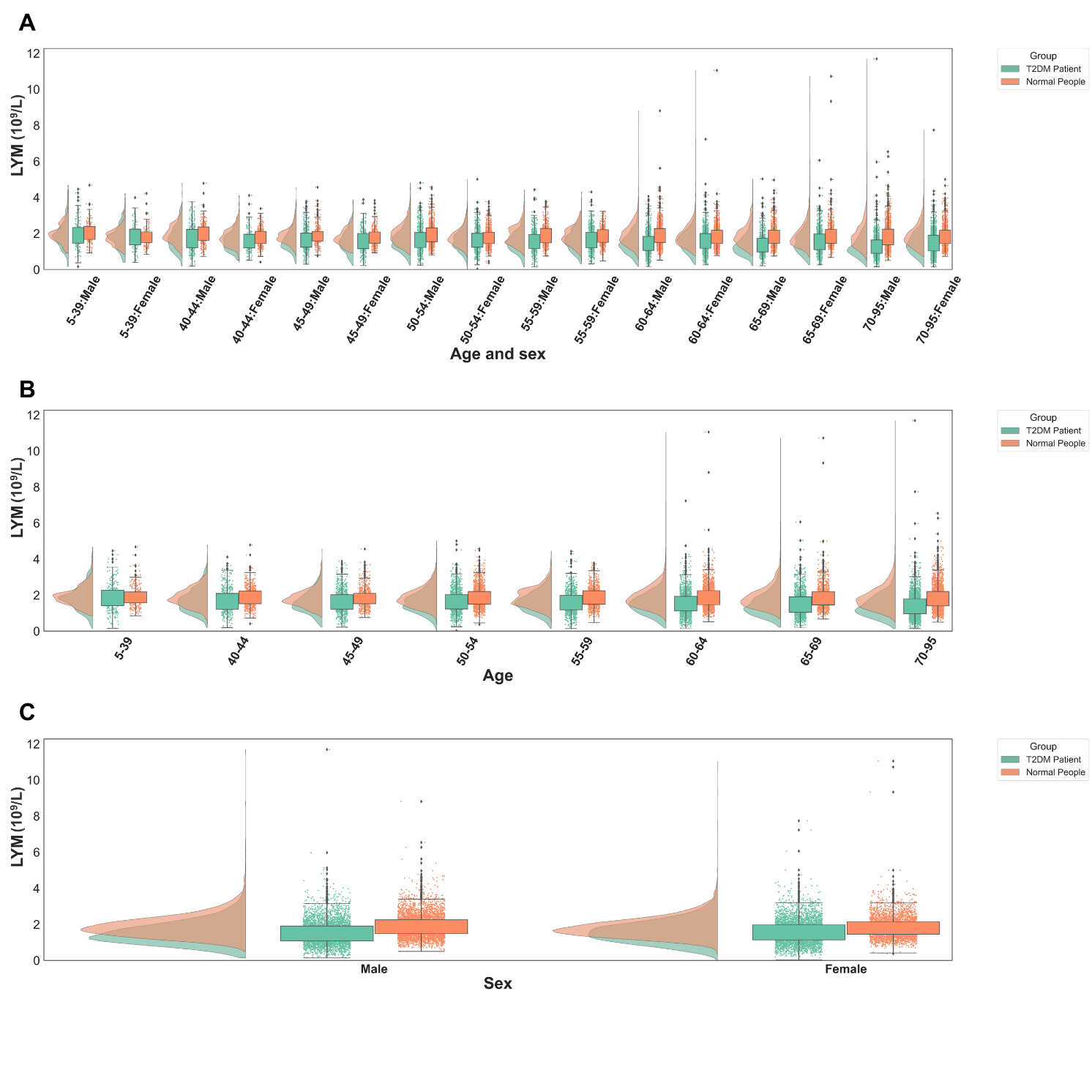
**

**Figure S10. Distribution of measured LYM values in different age and sex groups. (A)** Different age and sex groups. **(B)** Different age groups. **(C)** Different sex groups. All the LYM values were from origin EHR**.**

**
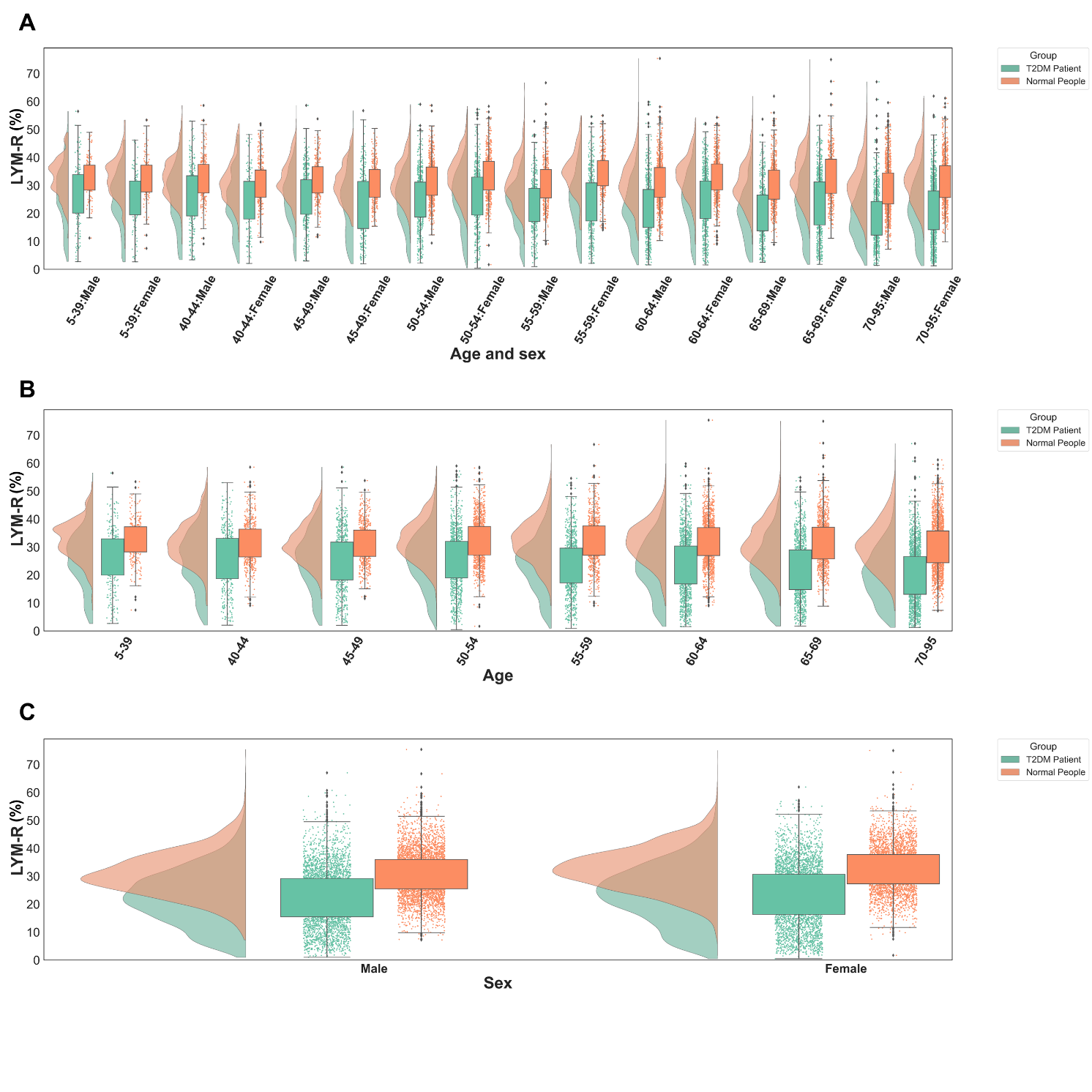
**

**Figure S11. Distribution of measured LYM-R values in different age and sex groups. (A)** Different age and sex groups. **(B)** Different age groups. **(C)** Different sex groups. All the LYM-R values were from origin EHR**.**

**
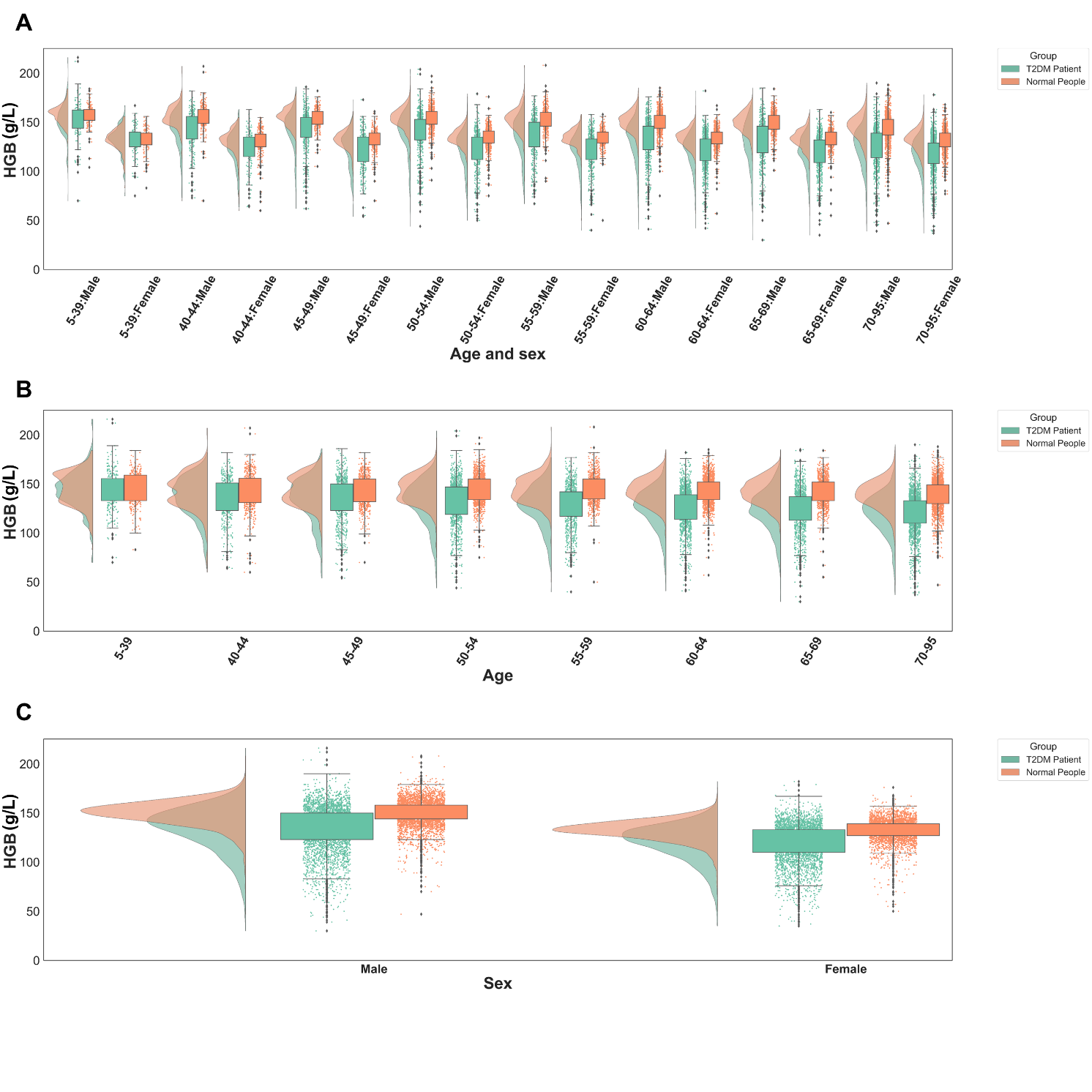
**

**Figure S12. Distribution of measured HGB values in different age and sex groups. (A)** Different age and sex groups. **(B)** Different age groups. **(C)** Different sex groups. All the HGB values were from origin EHR**.**

**
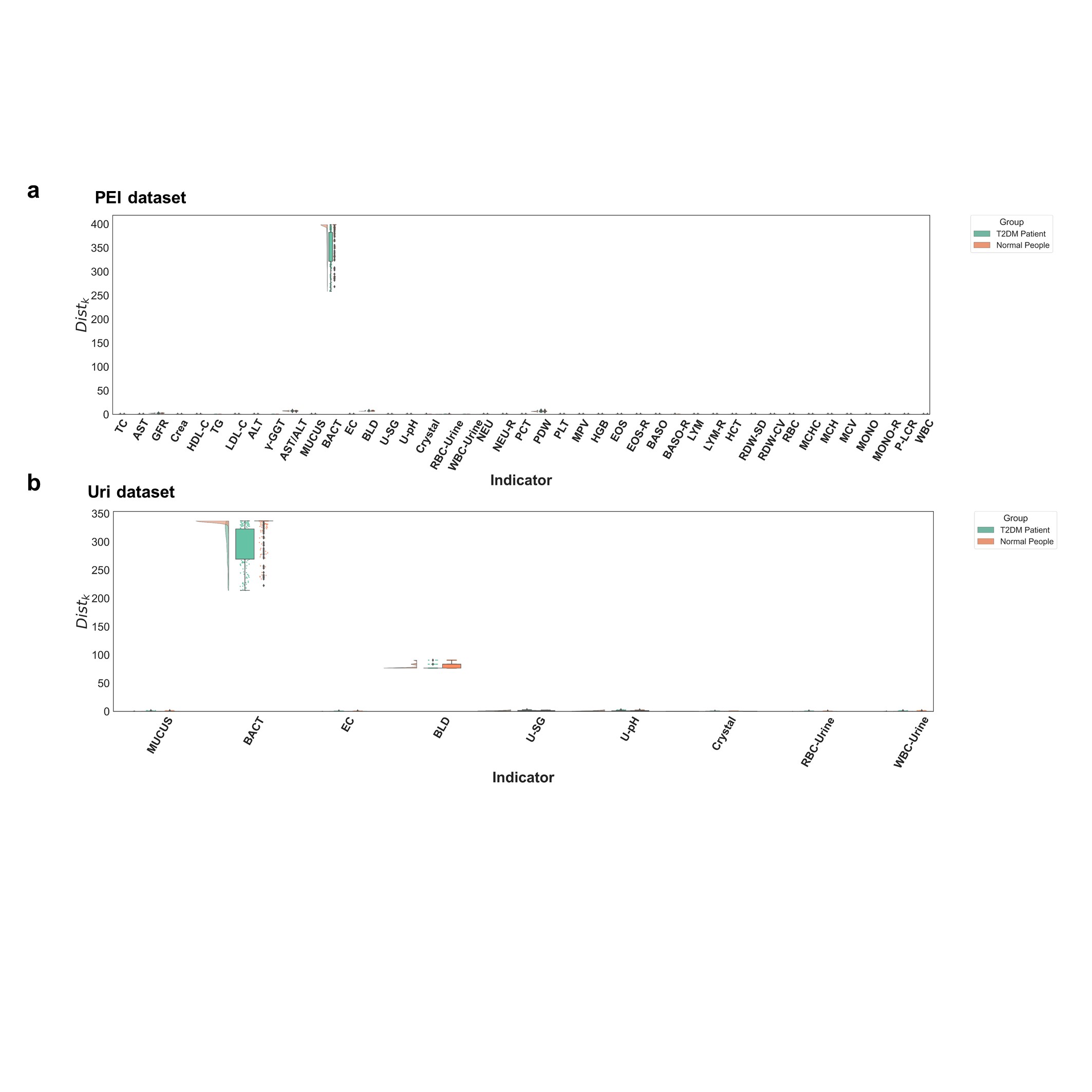
**

**Figure S13. Distribution of nine unbalanced indicators.** Both figures were plot when we built models using MVT-WDD-DI algorithm without balancing the deletion rate of each feature in positive and negative samples.

**
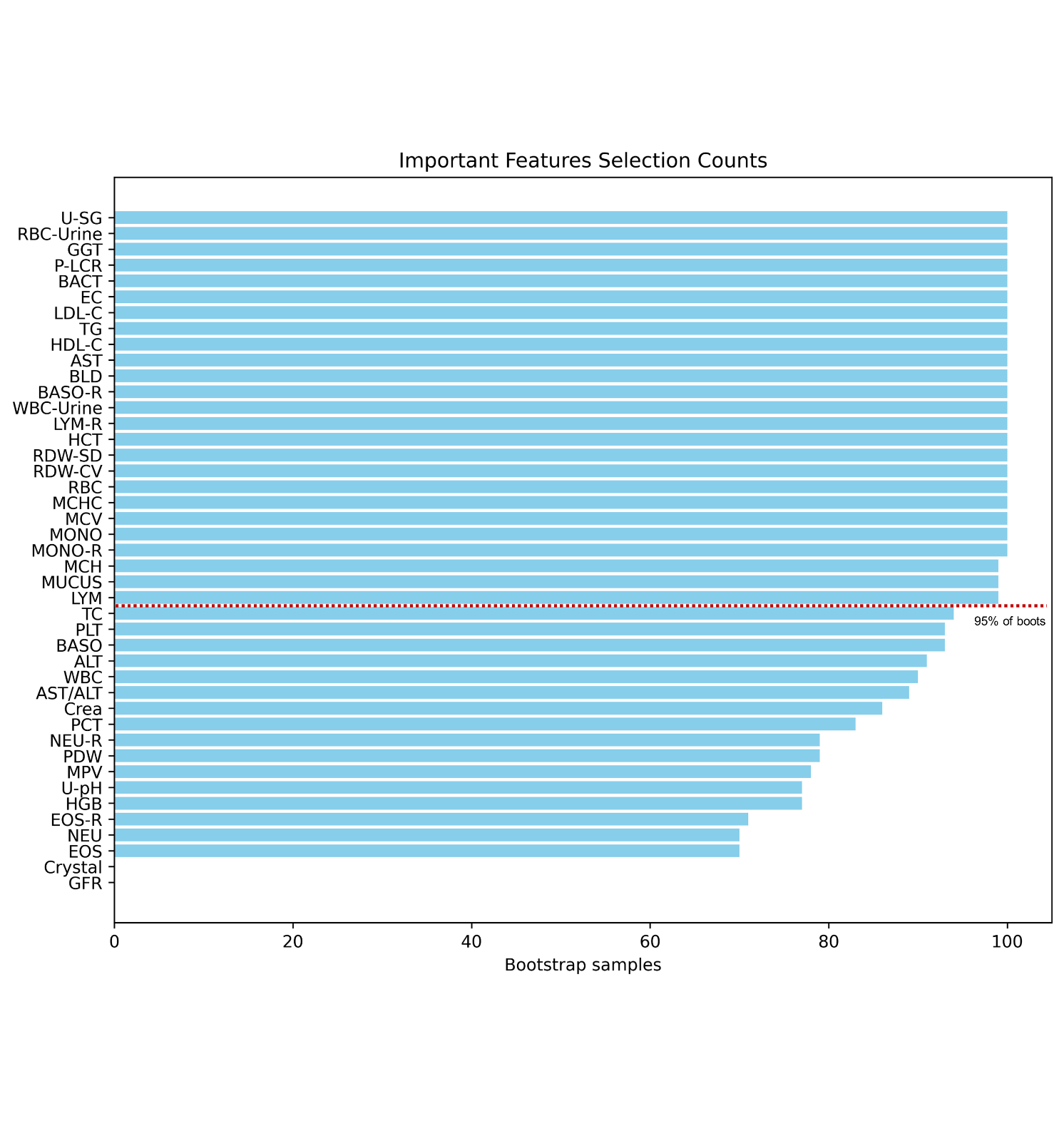
**

**Figure S14. Feature selection ranking of LASSO.**  Indicator ranking based on the number of times selected over bootstrap resampling during feature selection.


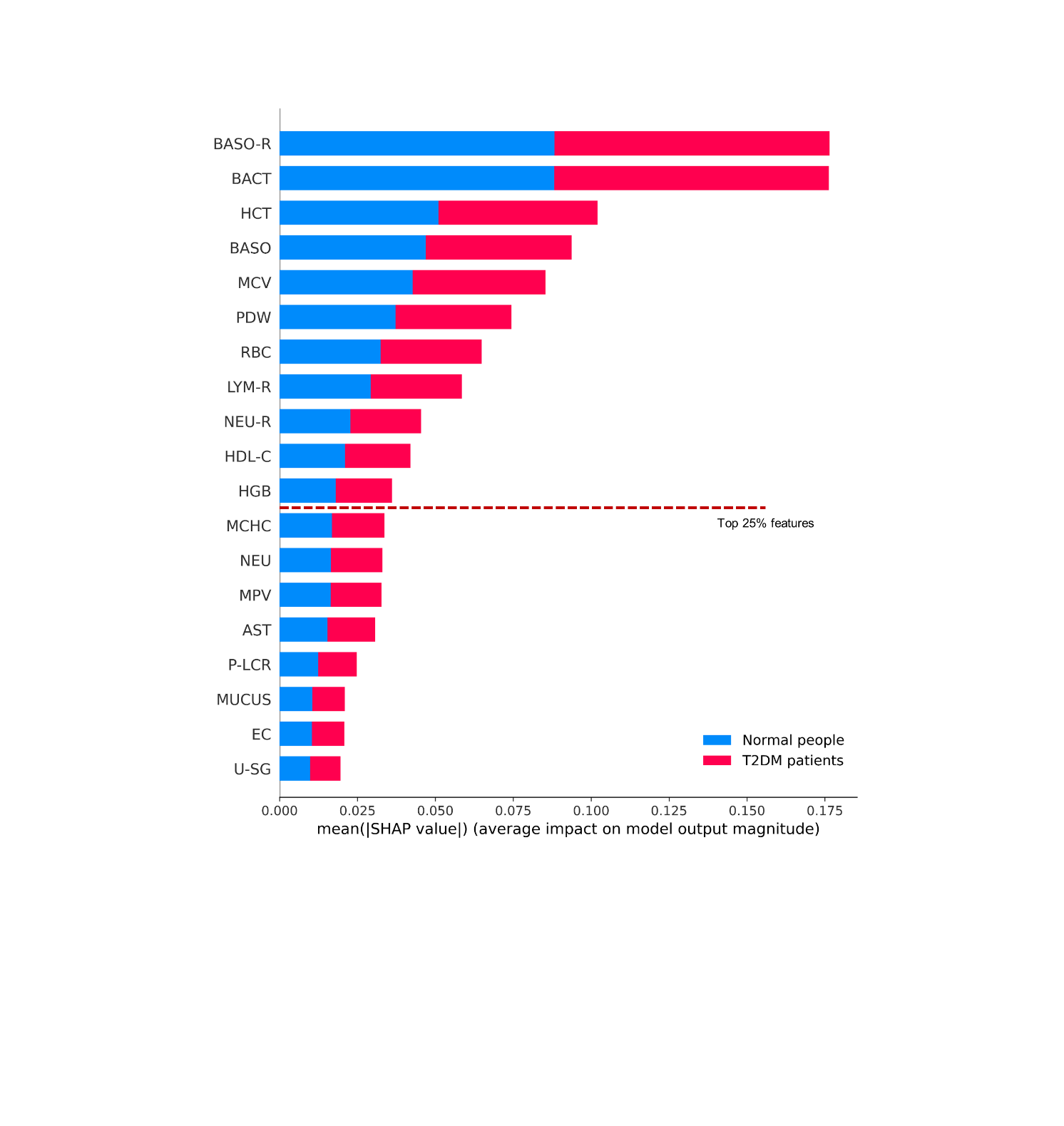


**Figure S15. Feature selection ranking of Shapley Additive exPlanations (SHAP) from random forest model.**  Indicator ranking based on the number of times selected over bootstrap resampling during feature selection.

Supplementary Table

**Table S1 The features used for modeling**

| **Name** | **Abbreviation** | **Physical examination item** |
| --- | --- | --- |
| Total Cholesterol | TC | Biochemical indicators |
| Aspartate Aminotransferase | AST |  |
| Glomerular Filtration Rate | GFR |  |
| Creatinine | Crea |  |
| High Density Lipoprotein Cholesterol | HDL-C |  |
| Triglyceride | TG |  |
| Low Density Lipoprotein Cholesterin | LDL-C |  |
| Alanine Aminotransferase | ALT |  |
| Gamma-Glutamyltransferase | γ-GGT |  |
| Aspartate Transaminase/Alanine Aminotransferase | AST/ALT |  |
| Mucinous Filament | MUCUS | Urinalysis indicators |
| Bacteria in Urine | BACT |  |
| Epithelial Cells in Urine | EC |  |
| Haematuria | BLD |  |
| Urine Specific Gravity | U-SG |  |
| Urine pH | U-pH |  |
| Crystallization | Crystal |  |
| Red Blood Cell in Urine | RBC-Urine |  |
| Leukocytes in Urine | WBC-Urine |  |
| Neutrophil | NEU | Blood Cell Analysis indicators |
| Neutrophil Rate | NEU-R |  |
| Plateletcrit | PCT |  |
| Platelet Distribution Width | PDW |  |
| Platelet | PLT |  |
| Mean Platelet Volume | MPV |  |
| Haemoglobin | HGB |  |
| Eosinophil | EOS |  |
| Eosinophil Rate | EOS-R |  |
| Basophil | BASO |  |
| Basophil Rate | BASO-R |  |
| Lymphocyte | LYM |  |
| Lymphocyte Rate | LYM-R |  |
| Haematocrit | HCT |  |
| Standard Deviation of Red Cell Distribution Width | RDW-SD |  |
| Coefficient Of Variation of Red Cell Distribution Width | RDW-CV |  |
| Red Blood Cell | RBC |  |
| Mean Corpuscular Haemoglobin Concentration | MCHC |  |
| Mean Corpuscular Haemoglobin | MCH |  |
| Mean Corpuscular Volume | MCV |  |
| Monocyte | MONO |  |
| Monocyte Rate | MONO-R |  |
| Platelet -Larger Cell Ratio | P-LCR |  |
| White Blood Cell | WBC |  |

**Table S2 The details of age and sex distribution for T2DM patients and normal people**

| **Number of T2DM patients** | | | |
| --- | --- | --- | --- |
| **Age group** | **Male** | **Female** | **Total by age** |
| 5-39 | 162 | 120 | 282 |
| 40-44 | 269 | 160 | 429 |
| 45-49 | 440 | 306 | 746 |
| 50-54 | 589 | 505 | 1094 |
| 55-59 | 439 | 509 | 948 |
| 60-64 | 626 | 769 | 1395 |
| 65-69 | 514 | 696 | 1210 |
| 70-95 | 824 | 1074 | 1898 |
| **Total by sex** | 3863 | 4139 | 8002 |
| **Number of normal people** | | | |
| **Age group** | **Male** | **Female** | **Total by age** |
| 5-39 | 147 | 135 | 282 |
| 40-44 | 274 | 280 | 554 |
| 45-49 | 301 | 320 | 621 |
| 50-54 | 662 | 608 | 1270 |
| 55-59 | 435 | 337 | 772 |
| 60-64 | 856 | 686 | 1542 |
| 65-69 | 598 | 465 | 1063 |
| 70-95 | 1137 | 761 | 1898 |
| **Total by sex** | 4410 | 3592 | 8002 |

**Table S3 The missing rate of each indicator in T2DM patients and normal people**

| **Missing rate (%)** | | | | | |
| --- | --- | --- | --- | --- | --- |
| **Indicator** | **T2DM patients** | **Normal people** | **Indicator** | **T2DM patients** | **Normal people** |
| **TC** | 8.34 | 0.12 | **PDW** | 4.89 | 0.06 |
| **AST** | 4.01 | 0 | **PLT** | 1.47 | 0 |
| **GFR** | 81.24 | 73.86 | **MPV** | 4.89 | 0.06 |
| **Crea** | 2.84 | 0 | **HGB** | 1.47 | 0 |
| **HDL-C** | 8.26 | 0.12 | **EOS** | 1.45 | 0 |
| **TG** | 8.29 | 0.12 | **EOS-R** | 1.42 | 0 |
| **LDL-C** | 8.26 | 0.12 | **BASO** | 1.44 | 0 |
| **ALT** | 4.02 | 29.52 | **BASO-R** | 1.42 | 0 |
| **GGT** | 3.87 | 1.46 | **LYM** | 1.46 | 0 |
| **AST/ALT** | 51.54 | 1.46 | **LYM-R** | 1.42 | 0 |
| **MUCUS** | 9.71 | 59.05 | **HCT** | 1.44 | 0 |
| **BACT** | 49.31 | 0.05 | **RDW-SD** | 1.45 | 0 |
| **EC** | 9.37 | 0 | **RDW-CV** | 1.59 | 0 |
| **BLD** | 84.95 | 0 | **RBC** | 1.47 | 0 |
| **U-SG** | 5.02 | 0 | **MCHC** | 1.42 | 0 |
| **U-pH** | 4.96 | 0 | **MCH** | 1.42 | 0 |
| **Crystal** | 9.45 | 0 | **MCV** | 1.42 | 0 |
| **RBC-Urine** | 6.99 | 0.01 | **MONO** | 1.45 | 0 |
| **WBC-Urine** | 10.72 | 0 | **MONO-R** | 1.42 | 0 |
| **NEU** | 1.49 | 0 | **P-LCR** | 6.15 | 0.06 |
| **NEU-R** | 1.42 | 0 | **WBC** | 1.54 | 0 |
| **PCT** | 4.89 | 0.06 |  |  |  |

**Table S4 The searching space for hyperparameter optimization**

|  | Hyperparameter | range | Final value |
| --- | --- | --- | --- |
| WDD-KNN | gamma | [2^-10^,2^10^] | 2^-3^ |
| MVT-WDD-DI | gamma |  | PEI dataset:2^0^ BCA dataset:2^2^ Uri dataset:2^0^ BioChem dataset:2^-2^ |
|  | delta |  | PEI dataset:2^-2^ BCA dataset:2^0^ Uri dataset:2^-2^ BioChem dataset:2^-2^ |
| MVT-WDD-BF | gamma |  | PEI dataset:2^3^ BCA dataset:2^1^ Uri dataset:2^1^ BioChem dataset:2^4^ |
|  | lambda |  | PEI dataset:2^5^ BCA dataset:2^4^ Uri dataset:2^4^ BioChem dataset:2^1^ |

**Table S5 The important features selected by at least one of the 8 models with AUC above 0.75**

| Neutrophil Rate |
| --- |
| Basophil Rate |
| Basophils |
| Lymphocyte Rate |
| Eosinophils |
| Mean Corpuscular Volume |
| Mean Corpuscular Haemoglobin |
| Coefficient Of Variation Of Red Cell Distribution Width |
| Haematocrit |
| Haemoglobin |
| Mean Platelets Volume |
| Plateletcrit |
| High Density Lipoprotein Cholesterol |
| Haematuria |
| Leukocytes in Urine |
| Mucinous Filament |
| Bacteria in Urine |
| Epithelial Cells in Urine |
| Urine pH |
| Urine Specific Gravity |

| **Feature** | **P significance** | **Feature** | **P significance** |
| --- | --- | --- | --- |
| TC | **** | PDW | **** |
| AST | **** | PLT | **** |
| GFR | ns | MPV | **** |
| Crea | ** | HGB | **** |
| HDL-C | **** | EOS | **** |
| TG | **** | EOS-R | **** |
| LDL-C | **** | BASO | **** |
| ALT | **** | BASO-R | **** |
| GGT | **** | LYM | **** |
| AST/ALT | **** | LYM-R | **** |
| MUCUS | **** | HCT | **** |
| BACT | **** | RDW-SD | **** |
| EC | **** | RDW-CV | **** |
| BLD | **** | RBC | **** |
| U-SG | *** | MCHC | **** |
| U-pH | **** | MCH | **** |
| Crystal | **** | MCV | **** |
| RBC-Urine | **** | MONO | **** |
| WBC-Urine | **** | MONO-R | **** |
| NEU | **** | P-LCR | **** |
| NEU-R | **** | WBC | **** |
| PCT | **** |  |  |
| ns: P value > 0.05, *: P value <= 0.05, **: P value <= 0.01, ***: P value <= 0.001 ****: P value <=0.0001 | | | |

**Table S6 The P significance of each indicator between T2DM patients and normal people using Mann-Whitney U test**

**Table S7. Performance of algorithms on PEI dataset when adding Body Mass Index (BMI): Mean (Standard)**

| **10-fold cross-validation** | | | | | |
| --- | --- | --- | --- | --- | --- |
| **PEI dataset (adding BMI)** | | | | | |
|  | AUC | ACC | Precision | Recall | F1 score |
| WDD-KNN | 0.9177(0.0031) | 0.8431(0.0037) | 0.8772(0.0048) | 0.7982(0.0067) | 0.8355(0.0042) |
| MVT-WDD-DI | 0.9144(0.0084) | 0.8420(0.0098) | 0.8742(0.0094) | 0.7992(0.0129) | 0.8347(0.0107) |
| MVT-WDD-BF | 0.8964(0.0087) | 0.8209(0.0088) | 0.8351(0.0089) | 0.8003(0.0122) | 0.8168(0.0096) |
| **Independent test** | | | | | |
| **PEI dataset (adding BMI)** | | | | | |
|  | AUC | ACC | Precision | Recall | F1 score |
| WDD-KNN | 0.9274(0.0089) | 0.8551(0.0108) | 0.8891(0.01502) | 0.8118(0.0177) | 0.8485(0.0117) |
| MVT-WDD-DI | 0.9203(0.0240) | 0.8494(0.0290) | 0.8874(0.0311) | 0.8004(0.0351) | 0.8414(0.0313) |
| MVT-WDD-BF | 0.9102(0.0171) | 0.8338(0.0250) (0.0235) | 0.8495(0.0250) | 0.8122(0.0266) | 0.8300(0.0198) |

Supplementary Reference

1. Vozarova B, Weyer C, Lindsay RS, Pratley RE, Bogardus C, Tataranni PA. High white blood cell count is associated with a worsening of insulin sensitivity and predicts the development of type 2 diabetes. *Diabetes* (2002) 51:455–461.

2. Twig G, Afek A, Shamiss A, Derazne E, Tzur D, Gordon B, Tirosh A. White blood cells count and incidence of type 2 diabetes in young men. *Diabetes care* (2013) 36:276–282.

3. Mangalesh S, Dudani S, Yadav P, Podury S. Evaluation of neutrophil-lymphocyte ratio in diabetes and coronary artery disease: a case control study from India. *American Heart Journal* (2021) 242:156–157.

4. Mzimela NC, Ngubane PS, Khathi A. The changes in immune cell concentration during the progression of pre-diabetes to type 2 diabetes in a high-fat high-carbohydrate diet-induced pre-diabetic rat model. *Autoimmunity* (2019) 52:27–36.

5. Reusch JEB. Diabetes, microvascular complications, and cardiovascular complications: what is it about glucose? *J Clin Invest* (2003) 112:986–988. doi: 10.1172/JCI19902

6. Fowler MJ. Microvascular and macrovascular complications of diabetes. *Clinical diabetes* (2011) 29:116–122.

7. Chu SG, Becker RC, Berger PB, Bhatt DL, Eikelboom JW, Konkle B, Mohler ER, Reilly MP, Berger JS. Mean platelet volume as a predictor of cardiovascular risk: a systematic review and meta-analysis. *Journal of Thrombosis and Haemostasis* (2010) 8:148–156. doi: 10.1111/j.1538-7836.2009.03584.x

8. Qian Y, Zeng Y, Lin Q, Huang H, Zhang W, Yu H, Deng B. Association of platelet count and plateletcrit with nerve conduction function and peripheral neuropathy in patients with type 2 diabetes mellitus. *Journal of Diabetes Investigation* (2021) 12:1835–1844. doi: 10.1111/jdi.13535

9. Tong PCY, Kong APS, So W-Y, Ng MHL, Yang X, Ng MCY, Ma RCW, Ho C-S, Lam CWK, Chow C-C, et al. Hematocrit, Independent of Chronic Kidney Disease, Predicts Adverse Cardiovascular Outcomes in Chinese Patients With Type 2 Diabetes. *Diabetes Care* (2006) 29:2439–2444. doi: 10.2337/dc06-0887

10. Hsieh Y-P, Chang C-C, Kor C-T, Yang Y, Wen Y-K, Chiu P-F. Mean Corpuscular Volume and Mortality in Patients with CKD. *CJASN* (2017) 12:237–244. doi: 10.2215/CJN.00970116

11. Swati S, Ramesh C. RED CELL DISTRIBUTION WIDTH IN PATIENTS WITH TYPE 2 DIABETES MELLITUS AND CORRELATION WITH MICROVASCULAR COMPLICATIONS. *AACE 28th Annual Scientific and Clinical Congress (AACE 2019)*. (2019)

12. Traveset A, Rubinat E, Ortega E, Alcubierre N, Vazquez B, Hernández M, Jurjo C, Espinet R, Ezpeleta JA, Mauricio D. Lower Hemoglobin Concentration Is Associated with Retinal Ischemia and the Severity of Diabetic Retinopathy in Type 2 Diabetes. *Journal of Diabetes Research* (2016) 2016:e3674946. doi: 10.1155/2016/3674946

13. Lee S-H, Kim H-S, Park Y-M, Kwon H-S, Yoon K-H, Han K, Kim MK. HDL-Cholesterol, Its Variability, and the Risk of Diabetes: A Nationwide Population-Based Study. *The Journal of Clinical Endocrinology & Metabolism* (2019) 104:5633–5641. doi: 10.1210/jc.2019-01080

14. Muller LMAJ, Gorter KJ, Hak E, Goudzwaard WL, Schellevis FG, Hoepelman AIM, Rutten GEHM. Increased Risk of Common Infections in Patients with Type 1 and Type 2 Diabetes Mellitus. *Clinical Infectious Diseases* (2005) 41:281–288. doi: 10.1086/431587

15. Maalouf NM, Cameron MA, Moe OW, Sakhaee K. Metabolic basis for low urine pH in type 2 diabetes. *Clinical Journal of the American Society of Nephrology* (2010) 5:1277–1281.

16. Eisner BH, Porten SP, Bechis SK, Stoller ML. Diabetic kidney stone formers excrete more oxalate and have lower urine pH than nondiabetic stone formers. *The Journal of urology* (2010) 183:2244–2248.

17. Bell DSH. Beware the low urine pH—the major cause of the increased prevalence of nephrolithiasis in the patient with type 2 diabetes. *Diabetes, Obesity and Metabolism* (2012) 14:299–303.

18. Akarsu E, Buyukhatipoglu H, Aktaran S, Geyik R. The Value of Urine Specific Gravity in Detecting Diabetes Insipidus in a Patient with Uncontrolled Diabetes Mellitus: Urine Specific Gravity in Differential Diagnosis. *Journal of General Internal Medicine* (2006) 21:C1–C2. doi: 10.1111/j.1525-1497.2006.00454.x

19. Guo X, Zhang S, Zhang Q, Liu L, Wu H, Du H, Shi H, Wang C, Xia Y, Liu X, et al. Neutrophil:lymphocyte ratio is positively related to type 2 diabetes in a large-scale adult population: a Tianjin Chronic Low-Grade Systemic Inflammation and Health cohort study. *European Journal of Endocrinology* (2015) 173:217–225. doi: 10.1530/EJE-15-0176

20. Shiny A, Bibin YS, Shanthirani CS, Regin BS, Anjana RM, Balasubramanyam M, Jebarani S, Mohan V. Association of Neutrophil-Lymphocyte Ratio with Glucose Intolerance: An Indicator of Systemic Inflammation in Patients with Type 2 Diabetes. *Diabetes Technology & Therapeutics* (2014) 16:524–530. doi: 10.1089/dia.2013.0264

21. Verma S, Husain M, Madsen C, Leiter LA, Rajan S, Vilsboll T, Rasmussen S, Libby P. Neutrophil-to-lymphocyte ratio predicts cardiovascular events in patients with type 2 diabetes: post hoc analysis of SUSTAIN 6 and PIONEER 6. *European Heart Journal* (2021) 42:ehab724.2479. doi: 10.1093/eurheartj/ehab724.2479

22. Carrasco-Zanini J, Pietzner M, Lindbohm JV, Wheeler E, Oerton E, Kerrison N, Simpson M, Westacott M, Drolet D, Kivimaki M, et al. Proteomic signatures for identification of impaired glucose tolerance. *Nat Med* (2022) 28:2293–2300. doi: 10.1038/s41591-022-02055-z

23. Zhao Y, Chaw JK, Ang MC, Daud MM, Liu L. A Diabetes Prediction Model with Visualized Explainable Artificial Intelligence (XAI) Technology. In: Badioze Zaman H, Robinson P, Smeaton AF, De Oliveira RL, Jørgensen BN, K. Shih T, Abdul Kadir R, Mohamad UH, Ahmad MN, editors. *Advances in Visual Informatics*. Lecture Notes in Computer Science. Singapore: Springer Nature (2024). p. 648–661 doi: 10.1007/978-981-99-7339-2_52

24. Shamai L, Lurix E, Shen M, Novaro GM, Szomstein S, Rosenthal R, Hernandez AV, Asher CR. Association of Body Mass Index and Lipid Profiles: Evaluation of a Broad Spectrum of Body Mass Index Patients Including the Morbidly Obese. *OBES SURG* (2011) 21:42–47. doi: 10.1007/s11695-010-0170-7
